# The metabesity factor HMG20A potentiates astrocyte survival and reactive astrogliosis preserving neuronal integrity

**DOI:** 10.7150/thno.57237

**Published:** 2021-05-12

**Authors:** Petra I. Lorenzo, Eugenia Martin Vazquez, Livia López-Noriega, Esther Fuente-Martín, José M. Mellado-Gil, Jaime M. Franco, Nadia Cobo-Vuilleumier, José A. Guerrero Martínez, Silvana Y. Romero-Zerbo, Jesús A. Perez-Cabello, Sabrina Rivero Canalejo, Antonio Campos-Caro, Christian Claude Lachaud, Alejandra Crespo Barreda, Manuel Aguilar-Diosdado, Eduardo García Fuentes, Alejandro Martin-Montalvo, Manuel Álvarez Dolado, Franz Martin, Gemma Rojo-Martinez, David Pozo, Francisco J. Bérmudez-Silva, Valentine Comaills, José C. Reyes, Benoit R. Gauthier

**Affiliations:** 1 Andalusian Center of Molecular Biology and Regenerative Medicine-CABIMER, Junta de Andalucía-University of Pablo de Olavide-University of Seville-CSIC, Seville, Spain.; 2 Unidad de Gestión Clínica Intercentros de Endocrinología y Nutrición, Instituto de Investigación Biomédica de Málaga (IBIMA), Hospital Regional Universitario de Málaga, Universidad de Málaga, Spain.; 3 Department of Normal and Pathological Histology and Cytology, University of Seville School of Medicine, Seville, Spain.; 4 University Hospital “Puerta del Mar”, Instituto de Investigación e Innovación en Ciencias Biomédicas de la Provincia de Cádiz (INiBICA), Cádiz, Spain.; 5 Endocrinology and Metabolism Department, University Hospital “Puerta del Mar”, Instituto de Investigación e Innovación en Ciencias Biomédicas de la Provincia de Cádiz (INiBICA), Cádiz, Spain.; 6 Unidad de Gestión Clínica de Aparato Digestivo, Hospital Universitario Virgen de la Victoria, Instituto de Investigación Biomédica de Málaga (IBIMA), Spain.; 7 Centro de Investigación Biomédica en Red de Diabetes y Enfermedades Metabólicas Asociadas (CIBERDEM), Madrid, Spain.

**Keywords:** HMG20A, metabolism, metabesity, astrocytes, inflammation, ORY1001.

## Abstract

**Rationale:** We recently demonstrated that the 'Metabesity' factor HMG20A regulates islet beta-cell functional maturity and adaptation to physiological stress such as pregnancy and pre-diabetes. HMG20A also dictates central nervous system (CNS) development via inhibition of the LSD1-CoREST complex but its expression pattern and function in adult brain remains unknown. Herein we sought to determine whether HMG20A is expressed in the adult CNS, specifically in hypothalamic astrocytes that are key in glucose homeostasis and whether similar to islets, HMG20A potentiates astrocyte function in response to environmental cues.

**Methods:** HMG20A expression profile was assessed by quantitative PCR (QT-PCR), Western blotting and/or immunofluorescence in: 1) the hypothalamus of mice exposed or not to either a high-fat diet or a high-fat high-sucrose regimen, 2) human blood leukocytes and adipose tissue obtained from healthy or diabetic individuals and 3) primary mouse hypothalamic astrocytes exposed to either high glucose or palmitate. RNA-seq and cell metabolic parameters were performed on astrocytes treated or not with a siHMG20A. Astrocyte-mediated neuronal survival was evaluated using conditioned media from siHMG20A-treated astrocytes. The impact of ORY1001, an inhibitor of the LSD1-CoREST complex, on HMG20A expression, reactive astrogliosis and glucose metabolism was evaluated *in vitro* and *in vivo* in high-fat high-sucrose fed mice.

**Results:** We show that *Hmg20a* is predominantly expressed in hypothalamic astrocytes, the main nutrient-sensing cell type of the brain. HMG20A expression was upregulated in diet-induced obesity and glucose intolerant mice, correlating with increased transcript levels of *Gfap* and *Il1b* indicative of inflammation and reactive astrogliosis. *Hmg20a* transcript levels were also increased in adipose tissue of obese non-diabetic individuals as compared to obese diabetic patients. HMG20A silencing in astrocytes resulted in repression of inflammatory, cholesterol biogenesis and epithelial-to-mesenchymal transition pathways which are hallmarks of reactive astrogliosis. Accordingly, HMG20A depleted astrocytes exhibited reduced mitochondrial bioenergetics and increased susceptibility to apoptosis. Neuron viability was also hindered in HMG20A-depleted astrocyte-derived conditioned media. ORY1001 treatment rescued expression of reactive astrogliosis-linked genes in HMG20A ablated astrocytes while enhancing cell surface area, GFAP intensity and STAT3 expression in healthy astrocytes, mimicking the effect of HMG20A. Furthermore, ORY1001 treatment protected against obesity-associated glucose intolerance in mice correlating with a regression of hypothalamic HMG20A expression, indicative of reactive astrogliosis attenuation with improved health status.

**Conclusion:** HMG20A coordinates the astrocyte polarization state. Under physiological pressure such as obesity and insulin resistance that induces low grade inflammation, HMG20A expression is increased to induce reactive astrogliosis in an attempt to preserve the neuronal network and re-establish glucose homeostasis. Nonetheless, a chronic metabesity state or functional mutations will result in lower levels of HMG20A, failure to promote reactive astrogliosis and increase susceptibility of neurons to stress-induced apoptosis. Such effects could be reversed by ORY1001 treatment both *in vitro* and *in vivo*, paving the way for a new therapeutic approach for Type 2 Diabetes Mellitus.

## Introduction

'Metabesity' is an emerging global term describing a wide range of diseases with underlying metabolic disorders and for which the etiology lies in complex interactions among genes and the obesogenic environment 1. Metabesity contributes to the generation of a chronic low-grade inflammation 2, which is recognized as the pathogenic link with Type 2 Diabetes Mellitus (T2DM). This inflammation targets peripheral tissues specialized in metabolism such as liver, adipose tissue and pancreatic islets and play a key role in insulin resistance and beta-cell dysfunction 3-5. Inflammation in the central nervous system (CNS) is an early event detected even prior to weight gain, and is likely implicated in the increased incidence of neurodegenerative diseases among T2DM patients 6. In fact, DM and neurodegenerative disease such as Alzheimer's disease (AD), share common elements such as insulin resistance, altered glucose metabolism, amyloid aggregation and inflammatory response 7. Accordingly, alterations in the insulin-central nervous system axis and brain glucose metabolism in DM and/or obese individual have been associated with the progression of AD 8, 9. Of particular interest, hypothalamic astrocytes, the major glucose metabolizers in the brain, were shown to couple CNS glucose uptake and nutrient availability via insulin signalling 9. Consequently, astrocytes within the hypothalamus, the hub of metabolic homeostasis, have come into the limelight as key regulator of appetite and energy expenditure.

Astrocytes are dispersed throughout the brain and regulate tissue homeostasis through structural and nutritive support of neurons, energy storage, and defense against oxidative stress. In view of their atypical morphology characterized by elongated processes of the cellular membrane (hence their name derived from star-like cells) that are in physical contact with other cell types and blood vessels, astrocytes facilitate synaptic transmission, neuronal plasticity and survival as well as to regulate blood flow relaying incoming peripheral signals to the CNS 2. As such astrocytes express a multitude of receptors for hormones and neuropeptides involved in metabolic control and accordingly to the registered signal will react by modulating the neuronal circuitry through increased transport of metabolic factors and release of growth factors, gliotransmitters, metabolites and cytokines 10. Notwithstanding to these physiological functions, astrocytes also react/adapt to CNS insults such as metabesity-induced neuroinflammation, through a complex and multifaceted process called reactive astrogliosis, characterized by cellular hypertrophy, increased proliferation and altered astrocyte functions 11, 12. Although reactive astrogliosis has an initial protective effect to preserve neuronal function and viability as well as repairing/healing of the initial damage, failure to resolve this reactive phenotype contributes to injury and to the development and progression of AD and DM 13, 14. Indeed, reactive astrogliosis impairs insulin signalling and glycogen storage, increases glucose uptake and glycolysis, and alters glucose transport in the brain 2. Although well characterized at the cellular level, the molecular mechanism controlling reactive astrogliosis remains ill-defined with only few factors such as ZEB2, an epithelial-to-mesenchymal transition (EMT)-related transcription factor or NF-kB, a pro-inflammatory transcriptional regulator which were found to promote reactive astrogliosis 15, 16. As such, identifying key regulators of astrocyte phenotypic state may prove valuable in the development of novel DM and AD theranostics.

Of particular interest is the chromatin remodeling factor HMG20A that we recently reported to be essential for pancreatic islet beta-cell functional maturation and adaptation to stress conditions such as hyperglycemia and pregnancy 17, 18. Consistent with these functional data small nucleotide polymorphisms (SNPs) in the *HMG20A* gene have been associated to T2DM as well as gestational diabetes mellitus (GDM) in Asian/Indian and European populations 19-25. HMG20A is also a key regulator of neuronal differentiation 26 by exerting global genomic changes through establishing active or silent chromatin 27. Mechanistically, HMG20A competes with HMG20B to relieve the transcriptional repression imposed by the complex LSD1-CoREST histone demethylase, which function is to silence neuronal and islet beta-cell genes through its interaction with the transcription factor REST 28-31. As HMG20A regulates the expression of key genes such as NEUROD, GK, and GLUTs common to both astrocytes and beta-cells we reasoned that this chromatin factor is a common master regulator of beta-cells and astrocytes, integrating inputs to altered glucose levels and stressful physiological conditions that dictate the phenotypic status of these cells 2. As such, herein, we explored whether HMG20A is expressed in astrocytes and whether it potentiates astrocytes function and phenotypic state in response to stress conditions. We also assessed whether HMG20A is expressed in human white adipose tissue (WAT) and serum and whether its levels correlate with obesity and/or T2DM. Finally, we evaluate the potential therapeutic benefits of ORY1001 (*aka*, Ladademstat), a potent inhibitor of the LSD1-CoREST complex that mimics the effects of HMG20A, to revert high fat diet-induced glucose intolerance and neuroinflammation. Our studies identify HMG20A as a core regulator of molecular pathways implicated in reactive astrogliosis a critical process for neuron survival in response to injury and/or metabolic stress. Accordingly, higher levels of HMG20A in WAT also correlated with a healthy status in obese individuals. Pharmacologically, reactive astrogliosis regressed in hypothalamus of high fat and high sucrose fed mice treated with ORY1001 correlating with improve glucose tolerance and lower HMG20A levels.

## Methods

### Ethical Statement

The collection of human samples was carried out in accordance with the Declaration of Helsinki (2008) of the Word Medical Association. Written informed consent was obtained from all participants. The study was approved by the Ethics and Clinical Investigation Committee of the Regional University Hospital of Malaga. All experimental mouse procedures were approved by either the Institutional Animal Care Committee of the Andalusian Center of Molecular Biology and Regenerative Medicine (CABIMER) or by the ethic committee of the University of Malaga, Biomedical Research Institute of Málaga (IBIMA) and performed according to the Spanish law on animal use RD 53/2013.

### Clinical Samples

Blood samples were selected from a cohort of the Pizarra Study 32. Briefly, the inclusion age was 18 to 65 years and individuals were excluded from the study if they were institutionalized for any reason, pregnant, or had a severe clinical or psychological disorder. Venous blood samples were collected in fasting conditions and stored in a biobank at the Regional University Hospital of Malaga (Spain). Subcutaneous adipose tissue biopsies were obtained from patients with morbid obesity who underwent bariatric surgery and from non-obese patients who underwent laparoscopic surgery for hiatus hernia at the Regional University Hospital of Malaga. Age and gender of patients in both samplings were not significantly different from the population distribution. Clinical characteristics are provided in **Table 1** and** Table 2**. The research was carried out in accordance with the Declaration of Helsinki (2008) of the Word Medical Association. Written informed consent was obtained from all participants. The studies were approved by the Ethics and Clinical Investigation Committee of the Regional University Hospital of Malaga.

### Mice

Animal studies were performed in compliance with the ARRIVE guidelines 33. C57BL/6J mice were purchased either from Charles Rivers (L'Arbresle Cedex, France) or Janvier Labs (Saint-Berthevin Cedex, France). Mice were housed in ventilated plastic cages and maintained on a 12-h light-dark cycle with ad libitum access to pellet chow and water. For studies on the impact of obesity on hypothalamic HMG20A expression, 8 weeks-old male mice were exposed, or not, to a high fat diet (HFD; D12451 Research Diets Inc, New Brunswick, NJ, USA), containing 45% of Kcal of saturated fat, for 15 weeks as previously described 34. Alternatively, for studies on the potential protective effect of the LSD1 inhibitor ORY1001 (Selleckchem, UK) on HFD-induced glucose intolerance and neuro-inflammation, a robust high fat 'Western-type' diet (50% of Kcal of saturated fat) combined with high sucrose (30% in the drinking water) *aka* HF-HS diet was supplied for 6 weeks to 15 weeks-old mice as previously described 35. Animals were treated or not daily with ORY1001 (0.0125 mg/kg body weight) starting at the same time as the HF-HS diet. Circulating glucose levels were measured from tail vein blood samples using an Optium Xceed glucometer (Abbott Cientifica SA, Barcelona, Spain). Oral glucose tolerance test (OGTT) were performed as previously described 34. In parallel, some mice were also used to extract the brain for immunofluorescence or RNA analysis.

### Primary hypothalamic astrocytes culture

Primary hypothalamic astrocytes were isolated and cultured as previously described 36, 37. Briefly, hypothalamus from 2 to 4-days-old C57BL/6J male mice were extracted under sterile conditions and triturated in Dulbecco's modified Eagle's Medium (DMEM) F-12 (Gibco, Thermo Fisher Scientific, MA, USA) containing 1% penicillin-streptomycin (Gibco). The suspension was centrifuged and the pellet resuspended in DMEM F-12 + 10% FBS + 1% antibiotics. Cells were grown in this culture media in T75 culture flasks at 37ºC and 5% CO_2_. The culture medium was refreshed every 2 days. At confluence, microglia and oligodendroglia were detached by shaking flasks at 220 rpm for 16 h at 37°C. The remaining attached astrocytes were then used for experiments.

### Primary hypothalamic neurons isolation and culture

Hypothalamic neurons were isolated from P2-P3 C57BL/6 mice male (procured from the University of Seville Animal Core Facility) following previously described protocols with some modifications 38, 39. Briefly, dissected hypothalami were minced and digested with papain (1 mg/mL) for 15 min under gentle agitation at 37ºC. After enzymatic digestion, hypothalami were subjected to mechanical disaggregation and passed through a 70 µm cell strainer. Neurons were then purified by an Optiprep gradient (Sigma-Aldrich) and cultured in Neurobasal medium (Thermo Fisher Scientifics) supplemented with 2% (w/v) B27 complement, 0.1% heat inactivated FBS, 125 nM L-glutamine and 50 μM beta-mercaptoethanol. After extraction, the culture was further treated with 2 μM arabinoside (Sigma-Aldrich) for 32 h to arrest growth of non-neuronal cells. Treatment with conditioned media from control, HMG20A-silenced astrocytes, or HMG20A-silenced astrocytes supplemented with ORY was performed at day 5 of culture for 24 h. In order to evaluate survival, the LIVE/DEAD Viability/Cytotoxicity assay for animal cells (Molecular Probes/ThermoFisher Scientific) was used according to the manufacturer's protocol. Images were acquired using an Imagexpress micro system and MetaXpress equipment (MDS Analytical Technologies). In order to determine the number of dead cells, EthD-1 positive cells were counted using ImageJ software.

### Primary spinal cord motoneurons isolation and culture conditions

Motor neuron cultures were prepared from embryonic 12.5-day spinal cords. Embryos were obtained from Friend leukaemia virus B (FVB) mice (University of Seville Animal Core Facility). Dissection of the spinal cord from embryonic (E12.5) were followed by the separation of neurons from the spinal cord tissue through mechanical and enzymatic cleavage as described 40, 41 with modifications. Briefly, spinal cords were dissected and meninges removed. Isolated spinal cords were transferred to ice cold Leibowitz's 15 medium (Thermo Fisher Scientifics) and fragmented. Spinal cord fragments were washed with HEPES buffer solution (137 mM NaCl, 2 mM KCl, 25 mM glucose, 25 mM HEPES buffer, pH 7.4, and 20 IU/mL penicillin plus 20 mg/mL streptomycin) and digested with 0.025% trypsin-EDTA (Gibco) for 8 min at 37ºC. After digestion, spinal cords were subjected to mechanical disaggregation by shaking in Leibowitz's L15 media containing 0.1 mg/mL DNase (Sigma-Aldrich). Additional disaggregation was carried out by careful pipetting samples in order to obtain a dispersion at single-cell level. Cells were purified after a 4% BSA gradient (Thermo Fisher Scientifics) by centrifugation at 180 *x*g for 5 min and resuspended in Leibowitz's 15 media. Next, cells were carefully layer over NycoPrep 1.077 gradient (Axis-shield) diluted with 50% of GHEBS buffer (137 mM NaCl, 2.7 mM KCl, 22.2 mM glucose, 25 mM HEPES buffer, pH 7.4, 20 IU/mL penicillin, 20 μg/mL streptomycin) and centrifugated at 520 *x*g for 10 min. Collected motor neurons were cultured in Neurobasal medium (Thermo Fisher Scientifics) supplemented with 2% (w/v) B27 complement, 2% (v/v) heat inactive horse serum, 125 nM L-glutamine, 50 μM beta-mercaptoethanol, 2 mM cytarabine and 10 ng/mL of CNTF, GDNF, BDNF and HGF. All factors were purchased from Peprotech (London, UK). The motor neuron culture media was changed every 48 hours, experimental conditions were carried out at day 5 of culture. To this end, media was complemented with increasing amounts of condition media obtained from astrocyte cultures silenced or not for HMG20A. In order to evaluate survival and measure cytotoxicity, a high throughput screening was carried out by using an Imagexpress micro system and MetaXpress equipment with a LIVE/DEAD Viability/Cytotoxicity assay for animal cells (Molecular Probes/ThermoFisher Scientific, Madrid, Spain). The numbers of dead cells were counted in 4 independent experiments.

### Astrocyte *in vitro* treatment

Primary hypothalamic astrocytes were cultured in the presence of: 1) low (3 mM), normal (17.5 mM) or high (30 mM) glucose concentrations for 1, 24 and 48 h, 2) 0.5 mM palmitate for up to 48 h or 3) 100 nM ORY1001 for 24, 48 and 72 h. These experiments were design as such that all samples were collected at the same final time point, including the control. Cells were then processed for either RNA extraction or immunofluorescence analysis.

### RNA interference

Primary astrocytes were transfected with either 50 μMol of a *HMG20A* small interfering (si)RNA or a luciferase siRNA (Sigma-Aldrich) as previously described 17. Cells were seeded at a density of either 2 x 10^5^ cells per well in 6-well plates or 2 x 10^4^ cells per well on glass cover slips in 24-well plates for RNA extraction or for immunofluorescence, respectively. Twenty-four h later cells were transfected with the siRNAs using Oligofectamine (Life Technologies) according to the manufacturer's instructions. Seventy-two h after transfection, media was collected for either measuring lactate release or for use as conditioned media while cells were processed for RNA isolation.

### RNA extraction and quantitative real-time PCR (QT-PCR)

Total RNA was extracted and purified from blood samples using the PAXgene Blood RNA Kit (PreAnalytiX GmbH, Hombrechtikon, CH) according to manufacturer's instructions. Qiagen RNeasy Mini and Micro kits (Qiagen, Madrid, Spain) were used for the extraction of total RNA from the brain, hypothalamus, white adipose tissue, pancreatic islets and astrocytes. Complementary DNA using 0.5 to 1 µg RNA was synthesized using the Superscript III Reverse Transcriptase (Invitrogen-Thermo Fisher Scientific, Madrid, Spain). The QT-PCR was performed on individual cDNAs using SYBR green (Roche) 42. Gene-specific primers were designed using Primer3Web (https://primer3.ut.ee/) and the sequences are listed in **Table S1**. Expression levels were normalized to various housekeeping genes including *β-ACTIN*, *HISTONE*, *Cyclophilin* and *Gapdh.* The relative gene expression was calculated using the 2^-ΔΔCt^ method 17.

### RNA-Sequencing

Quality of total RNA extracted from primary hypothalamic astrocytes treated or not with siHMG20A was verified using nanoRNA kit on the Agilent 2100 bioanalyzer. Quantification of RNA was evaluated by Qubit (Qubit™ RNA HS Assay). Purified RNA (150 ng) with RNA integrity number higher than 7 was used for library preparation using the TruSeq Stranded mRNA Library Preparation Kit (Illumina, San Diego, CA, USA) and sequenced on a NextSEq500 (Illumina). Two biological replicates per condition were sequenced. A mean of 30 million paired-end reads of 75 bp were generated for each sample.

RNA-seq data were initially filtered using the FASTQ Toolkit (v1.0.0) program. RNA-seq reads were aligned to the mouse reference genome mm9 using subjunc function from Rsubread package (v1.28.1) with TH1=2 and unique=TRUE parameters. The downstream analysis was performed on bamfiles with duplicates removed using the samtools (v0.1.19) rmdup command. FeatureCounts function from Rsubread package was used to assign reads to UCSC mm9 KnownGenes (miRNAs were discarded from the analysis) using GTF.featureType=“exon”, GTF.attrType=“gene_id”, isPairedEnd = TRUE and strandSpecific = 2 parameters on duplicate removed bamfiles. Then differential gene expression analysis was performed using the voom/limma (v.3.34.9) and edgeR (v.3.20.9) Bioconductor packages. Genes that were expressed at ≥ 1 counts per million counted reads in all samples were analyzed, resulting in 12632 genes out of 21761 UCSC KnownGene genes. CalcNormFactors() function using TMM method was used to normalize samples. An adjusted *p*-value < 0.05 was determined as threshold for multiple comparisons.

Pathway analysis was performed using Gene Set Enrichment Analysis (GSEA). First LCPM (log2(CPM)) was calculated for all 12632 expressed genes for each replicate. Z-Score matrix was created and GSEA (v.2.0) analysis was performed with 1000 permutations. Normalized enrichment score (NES) and false discovery rate (FDR) were used to quantify enrichment magnitude and statistical significance, respectively. Protein interaction analysis using annotated protein-coding genes was performed using STRING 43.

### Lactate concentrations in the culture media

Lactate levels were measured in the media of primary hypothalamic astrocyte cultures 72 h after siCT or siHMG20A transfection. One mL of medium was removed from each culture and lyophilized. Secreted lactate levels were determined using a Lactate assay kit following the manufacturer's instructions (EnzyChrom, BioAssay Systems, Hayward, CA, USA).

### Cell death and mitochondrial metabolism

Cell death (apoptosis) was measured using the Cell Death Detection ELISA kit (Roche Diagnostics, Madrid, Spain) while mitochondrial metabolism was assessed using the MTT assay, according to the manufacturer´s recommendations (Roche, Spain). A Seahorse Cell Mito Stress Test recorded using a XF24 Extracellular Flux Analyzer (Agilent) was performed, as previously described to measure mitochondrial bioenergetics in primary astrocytes silenced or not with siHMG20A (5000 cells/well) 44. At the end of the measurement, cell protein content was determined to normalize the data.

### HMG20A ELISA

Secreted HMG20A levels were measured by ELISA, following the manufacturer's instructions (MyBioSource, San Diego, CA, USA). The sensitivity of the method was 1 ng/mL. Absorbance in each well was measured by using a Varioskan Flash Spectral Scanning Multimode (ThermoFisher Scientific) and serum HMG20A concentrations were calculated from the standard curve. Samples were run in duplicates.

### Immunofluorescence studies

For brain immunostaining, mice were transcardially perfused with 4% PFA in 0.1M PB and, after overnight post fixation in the same fixative, brains were coronally sectioned into six series of 50 μm slices using a vibratome (Leica). Alternatively, brains were embedded in paraffin and sectioned at 6 μm using a microtome. Free floating brain sections and fixed astrocyte cultures were immunostained with the following antibodies (**Table S2**): Mouse anti-NEUN, mouse anti-GFAP, mouse anti-OLIG2, rabbit anti-IBA1 and rabbit anti-HMG20A or a combination of these antibodies. Secondary antibodies used were (**Table S2**): Rhodamine Red anti-rabbit, Fluorescein anti-mouse, Alexa Fluor 488 anti-mouse and Alexa Fluor 568 anti-rabbit. Of note, antibodies against HMG20A and IBA1 were both generated in rabbit. As such the immunostainings were performed sequentially, first with the anti-HMG20A antibody and the secondary Alexa anti-rabbit 568 antibody, and after blocking, incubation with the anti-IBA1 antibody and the secondary Alexa anti-rabbit 488 antibody. As negative controls primary antibodies were omitted from the reaction in the presence of all the reagents and secondary antibodies. All the negative controls were run in parallel for each immunodetection assay and always resulted in lack of signal (data not shown). Nuclei were stained with 0.0001% of 4′,6-diamidino-2-phenylindole (DAPI, Sigma-Aldrich). Epifluorescence microscopy images were acquired using either a Leica DM6000B or an Olympus X71 fluorescence microscope.

### Morphometry

To assess ORY1001-mediated reactive astrogliosis, images of GFAP immunostained astrocytes treated or not with the drug were acquired from 5 independent areas of each slides using a 10X objective. Each experimental condition was performed in triplicate in 3 independent astrocyte primary cultures. Images were then analyzed using the ImageJ software by delineating edges of GFAP^+^ cells and calculating the surface area (μm^2^) of at least *46-56* images from which the area of at least 40 GFAP^+^ cells were measured. GFAP signal intensity was assessed in 337-396 cells.

### Western Blotting

Primary astrocyte cultures as well as brain samples were processed and protein concentration determined as previously reported 37. Western blotting was performed as previously described 44. Antibodies and dilutions employed are provided in **Table S2**.

### Statistical Analysis

Measurements of the data were made in at least 4 independent samples. Results are presented as means ± SEM. Statistical comparisons were performed by either the Student's *t*-test or ANOVA and all *p* values less than or equal to 0.05 were considered statistically significant. Statistical analyses were performed using either the SPSS software version 18.0 (IBM, NY, USA; clinical study) or the GraphPad Prism software version 8 (GraphPad Software, La Jolla, USA).

### Data and Resource Availability

All data generated and/or analyzed in the context of the current study are included in the published article and Supplemental Material. The RNA-seq raw data that support the findings of this study have been deposited in the Gene Expression Omnibus repository with accession number GSE161334.

## Results

### HMG20A is expressed in hypothalamic astrocytes

**Figure 1:**
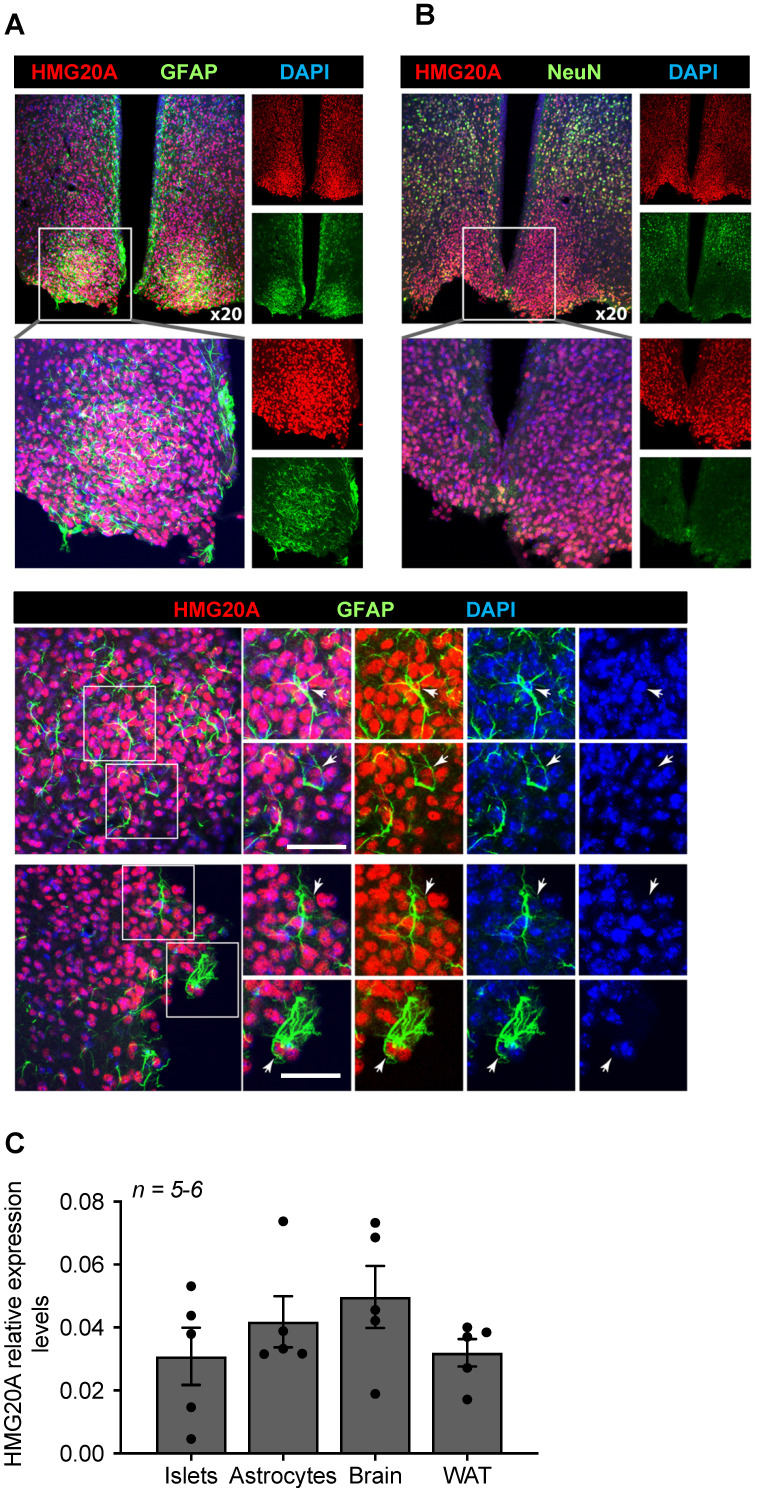
** HMG20A is expressed in hypothalamic astrocytes.** (**A**) Representative confocal microscopy images of free-floating mouse brain sections immunostained for HMG20A (red) and GFAP, an astrocyte marker (green, upper panel left) or (**B**) NeuN a neuronal marker (green, upper panel right). Nuclei were stained using DAPI (blue). The bottom panel in (**A**) provides higher magnification images for the detection of HMG20A (red) and GFAP (green) co-expression in hypothalamic astrocytes. Arrows point to astrocytes expressing HMG20A. White bar corresponds to 50 μm. (**C**) Relative expression levels of *Hmg20a* in mouse islets, astrocytes, brain and white adipose tissue (WAT). *Hmg20a* transcript levels were normalized to those of the housekeeping gene *Gapdh* and/or *Cyclophilin*. *n = 5-6* biological replicates analyzed in duplicate.

Transcript levels for *Hmg20a* were previously shown by *in situ* hybridization to be highly abundant in the outer cortex of the developing mouse brain 26 and postnatally in the dentate gyrus and pyramidal layer of the hippocampal formation (https://mouse.brain-map.org/experiment/ivt?id=357093&popup=true) 45. Notwithstanding, the expression of the protein and cell-type distribution within the mouse adult brain has yet to be mapped. To address this point, immunofluorescence studies using a validated anti-HMG20A polyclonal antibody 17 were performed on various region of the mouse brain, focusing on its expression in astrocytes and neurons. HMG20A was detected in various area of the brain, mainly co-localizing with a subpopulation of NEUN^+^ neurons (**Figure S1**). In addition, HMG20A also colocalized with GFAP^+^ astrocytes (**Figure S2**) including the hypothalamic region with preponderance within the arcuate and ventromedial nuclei (**Figure 1A and B**). In contrast, HMG20A did not co-localize with either OLIG2 or IBA1 indicating exclusion of this factor from oligodendrocytes and microglia (**Figure S3** and **S4**). The expression pattern was confirmed at the transcript level in isolated hypothalamic astrocytes (**Figure 1C**). Interestingly, *Hmg20a* expression in these astrocytes was comparable to that of whole brain, islets and white adipose tissue (WAT) 17. As the mean ratio of astrocytes, that comprise 20% of the total glial cell population, to neurons was recently estimated to be approximately 0.18 in the brain 46, 47, our data suggest that HMG20A is highly expressed in hypothalamic astrocytes while neurons likely express lower levels throughout the brain.

### Metabesity induces Hmg20a gene expression in the mouse hypothalamus as well as in human adipose tissue

**Figure 2:**
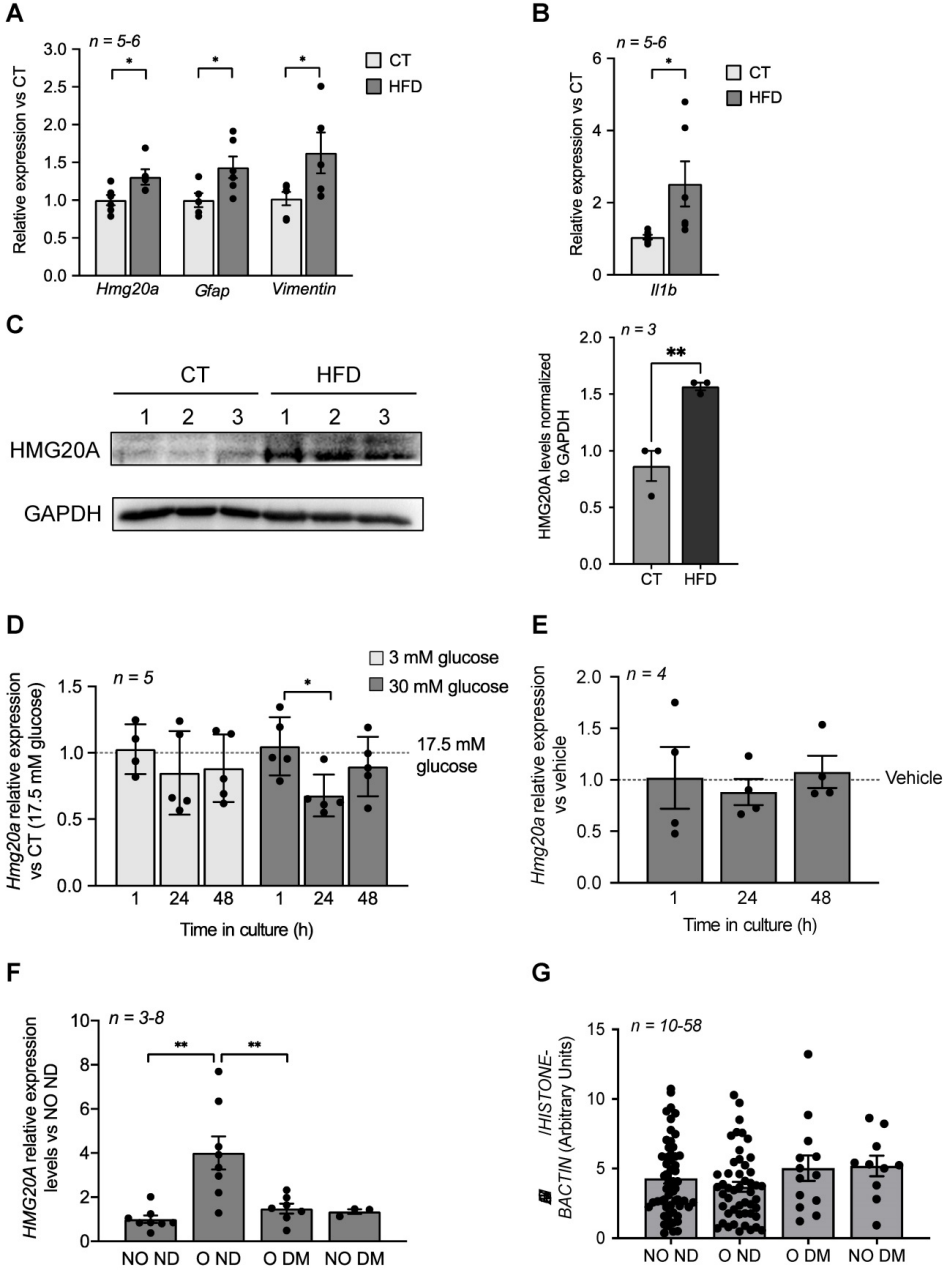
** HMG20A levels are increased by physiological/metabolic stresses in both mouse and human.** (**A**) Relative expression levels of *hmg20a*, *Gfap*, *Vimentin* and (**B**) *Il1b* in hypothalamus extracted from mice fed either a normal diet (CT) or a high fat diet (HFD). Transcript levels were normalized to those of the housekeeping gene b-Actin. Values are referred to the average expression levels detected in CT mice. *n = 5-6* biological replicates analyzed in duplicate. *p < 0.05, unpaired *t*-test CT versus HFD. (**C**) HMG20A protein levels (left panel) and quantification (right panel) in hypothalamus extracted from mice fed either a normal diet (CT) or a high fat diet (HFD). Each lane represents a protein extract of one hypothalamus isolated from either CT or HFD mouse. GAPDH was used as the housekeeping protein for normalization. **p < 0.01, unpaired *t*-test CT versus HFD. Relative expression levels of *Hmg20a* in isolated mouse astrocytes cultured in either (**D**) increasing concentration of glucose or (**E**) 0.5 mM palmitate for 1, 24 and 48 h. Transcript levels were normalized to those of the housekeeping gene *Gapdh* and/or *Cyclophilin.* Values are referred to the average expression levels detected in astrocytes cultured either in 17.5 mM glucose (**D**) or untreated (vehicle, **E**). *n = 4-5* biological replicates analyzed in duplicate. *p < 0.05, unpaired *t*-test 1 versus 24 h. (**F**) *HMG20A* expression levels were measured in subcutaneous white adipose tissue biopsies that were obtained from non-obese/non-diabetic (NO ND), obese/non-diabetic (O ND), obese/diabetic (O DM) and non-obese/diabetic (NO DM). The expression of *HMG20A* was corrected to those of the housekeeping gene *HISTONE*. Values are referred to the average expression detected NO ND individuals. *n = 3-8* individuals per group, analyzed in duplicate. ** p<0.01, unpaired *t-*test NO ND versus O ND or O DM. (**G**) Leukocytes were isolated from blood samples extracted from non-obese/non-diabetic (NO ND), obese/non-diabetic (O ND), obese/diabetic (O DM) and non-obese/diabetic (NO DM) and relative expression levels of *HMG20A* were assessed by quantitative PCR. The expression of HMG20A was corrected to those of the housekeeping genes *HISTONE* and *B-ACTIN*. *n = 10-58* individuals per group analyzed in duplicate.

We previously demonstrated that *Hmg20a* expression is enhanced in pancreatic islet beta-cells in response to increased insulin demands such as during pregnancy or pre-diabetic conditions correlating with sustained normoglycemia 17. The hypothalamus is also a nexus in the control of glucose homeostasis via the arcuate nucleus that, in part, fine tunes insulin secretion from beta-cells while the ventromedial nucleus favours the counter regulatory response through potentiating glucagon release from alpha-cells 48. As *Hmg20a* was found to be highly expressed in hypothalamic astrocytes, we reasoned that it may play a similar role to that observed in beta-cells and facilitate the neuronal response and/or survival to metabesity conditions. To this end, we assessed whether *Hmg20a* expression levels were altered in the hypothalamus of a high fat diet (HFD) induced-obesity and pre-diabetic mouse model that we recently developed 34. Transcript levels of *Hmg20a* along with those of the astrocytic markers *Gfap* (glial fibrillary acidic protein) and *Vimentin* were significantly increased in the hypothalamus of HFD fed mice as compared to those fed a normal chow diet (**Figure 2A**). Consistent, with obesity-induced neuroinflammation, expression of *Il1b* was higher in HFD treated mice as compared to normal chow fed mice (**Figure 2B**). A concomitant increase in HMG20A protein levels of approximately 1.6-fold was also observed in HFD fed mice (**Figure 2C**). Hyperglycemia and hyperlipidemia are hallmarks of metabesity 2. As such, we sought to determine the direct contribution of either high glucose or palmitate on expression levels of *Hmg20a* in isolated hypothalamic astrocytes. High glucose transiently decreased *Hmg20a* expression at 24 h post treatment whereas palmitate did not prompt changes in expression of the factor (**Figure 2D and E**) suggesting that glucose but not lipids may modulate expression of *Hmg20a*.

We previously demonstrated that *HMG20A* expression levels are blunted in islets isolated from human T2DM donors 17. We therefore contemplated whether *HMG20A* expression is increased as a tissue adaptive response to glucose in order to enhance cell function, as assessed by higher levels in brain of prediabetic mice or islet beta-cells of gestating female but collapses in chronic pathophysiological conditions such as DM, resulting in cellular disarray. To examine this possibility in humans, we assessed the expression levels of *HMG20A* in subcutaneous adipose tissue biopsies taken from donors who were: 1) non-obese/non-diabetic (NO ND), 2) obese/non-diabetic (O ND), 3) obese/diabetic (O DM) and 4) non-obese/diabetic (NO DM) (**Table 2**). We elected for WAT as *HMG20A* is expressed in this glucose-homeostasis regulatory tissue (**Figure 1C**) for which biopsies can be easily acquired as compared to the brain. Consistent with our premise, *HMG20A* expression levels were significantly higher in WAT of obese/non-diabetic as compared to either non-obese non-diabetic or diabetic individuals independent of weight (**Figure 2F**). In order to extend these findings as well as to identify a possible blood diagnostic marker for DM, HMG20A levels were also evaluated in blood serum and leukocytes of healthy or diabetic donors (**Table 1**). No significant differences in protein or transcript levels were detected in serum or leukocytes among the various groups (**Figure S5 and Figure 2G**). In summary, HMG20A expression is modulated in mouse hypothalamus and human WAT in response to metabesity conditions suggesting a potential adaptive mechanism similar to pancreatic islets 17.

### HMG20A regulates pathways involved in reactive astrogliosis

**Figure 3:**
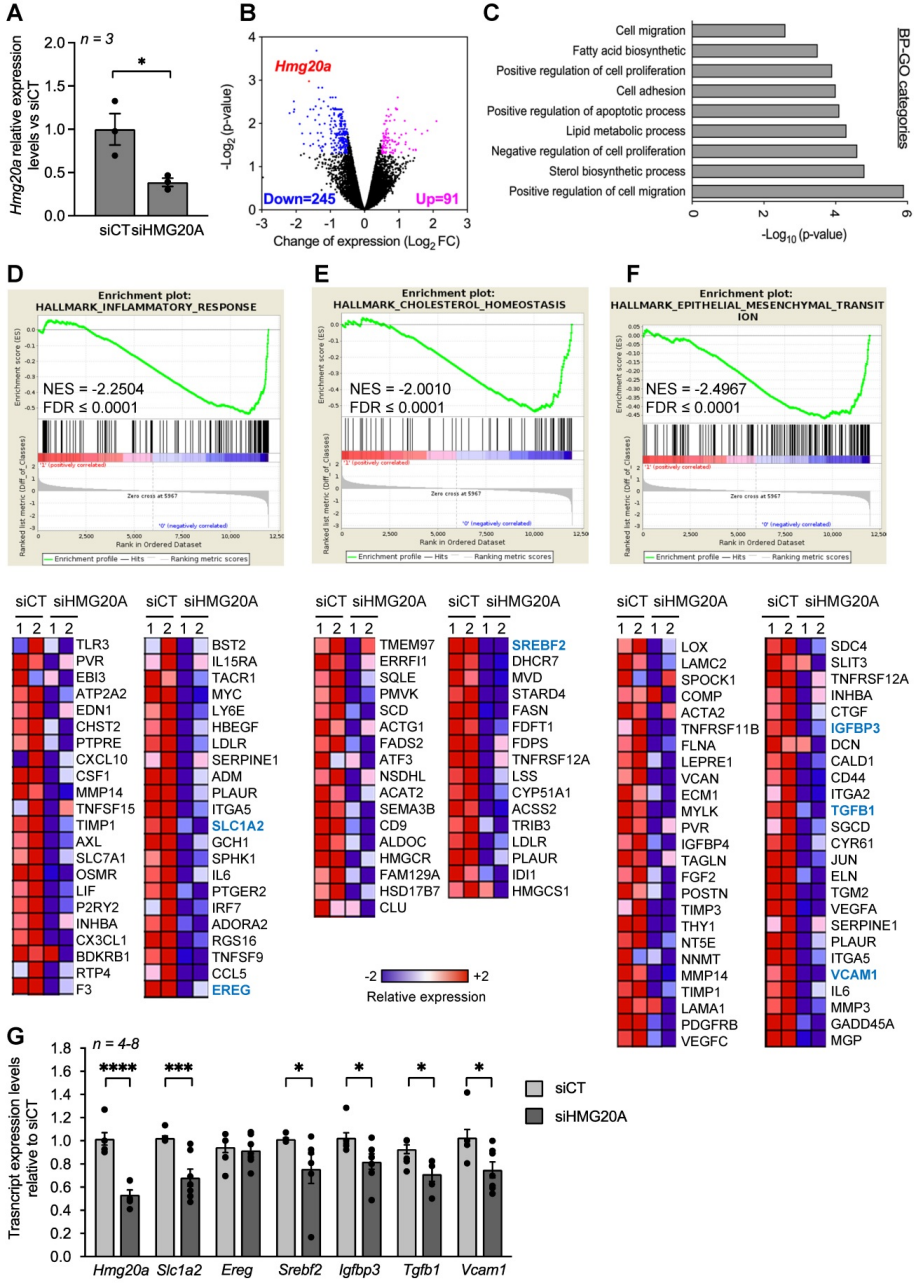
** HMG20A silencing represses signaling pathways linked to reactive astrogliosis.** (**A**) Relative expression levels of *Hmg20a* in astrocytes exposed to either a control luciferase siRNA (siCT) or a siRNA targeted to HMG20A (siHMG20A). Transcript levels were normalized to those of the housekeeping gene *Gapdh* and/or *Cyclophilin*. Values are referred to the average expression levels detected in siCT. *n = 3* biological replicates of primary astrocyte preparations pooled from 3-6 mice and analyzed in duplicate. *p < 0.05, unpaired *t*-test siCT versus siHMG20A. (**B**) Volcano plot representation of differentially regulated genes after HMG20A silencing, analyzed by RNA-seq. Genes significantly (*p-value < 0.05*) downregulated are marked in blue (Log2FC ≥ - 0.5) and the upregulated ones in purple (Log2FC ≥ 0.5). *Hmg20A* is highlighted as a red dot. (**C**) Biological process (BP)-GO categories enriched after HMG20A silencing, analyzed using the David software. (**D**), (**E**) and (**F**) Representative enriched pathways (upper panels) related to reactive astrogliosis. The analysis was performed using the GSEA software. Normalized Enrichment Score (NES) and False Discovery Rate (FDR) are indicated within the plots. Lower panels depict heatmaps of the relative expression of those genes significantly downregulated after HMG20A silencing in each enriched pathways in two independent RNAseq. (**G**) Confirmatory QT-PRC of selected target genes from the various pathways altered by HMG20A repression. Transcript levels were normalized to those of the housekeeping gene *Gapdh* and/or *Cyclophilin*.

In order to decipher the regulatory function of HMG20A in astrocytes, the chromatin factor was silenced and RNA-seq was carried out to identify differentially expressed genes (DEGs) as well as their downstream functional consequences. A 60% repression was achieved using a previously validated siHMG20A when compared to a control luciferase siRNA (siCT) (**Figure 3A**) 17. Transcriptome analysis revealed that 91 transcripts were upregulated and 245 were downregulated in siHMG20A transfected astrocytes as compared to siCT astrocytes (**Figure 3B**). Integrated bioinformatic tools using different algorithms were applied to analyse DEGs in order to reveal key pathways altered by HMG20A repression. Several gene ontology (GO) biological process (BP) related to cell migration and adhesion as well as to fatty acid and sterol biosynthetic processes were down-regulated subsequent to HMG20A depletion in astrocytes (**Figure 3C**). Interestingly, most DEGs within these BPs were down-regulated as exemplified by genes implicated in the steroid biosynthesis pathway (**Figure S6**). This premise was reinforced using an unbiased STRING analysis that revealed 4 main biological clusters of down-regulated genes (stress response and inflammation, growth factors and signal transductions, cytoskeleton-integrin connection and lipid metabolism) while none were found for up-regulated genes (**Figure S7**). The GSEA algorithm was next applied in order to gain further insight into the functional clustering of DEGs. In line with both GO and STRING, GSEA revealed common functional groups including inflammatory response, cholesterol homeostasis and epithelial to mesenchymal transition (EMT) (**Figure 3D-F**) as well as several cytokine related pathways such as TGFB1 and IL2 (**Figure S8**) that were down regulated by siHMG20A. Of interest, these pathways and associated DEGs (heatmaps, **Figure 3D-F**) have been linked to reactive astrogliosis and neuron viability. For example, TGFB1 was shown to be both a para- and auto-crine signalling molecule that stimulates the reactive astrocyte phenotypic state while VCAM1 and IGFBP3 facilitate EMT, a cellular process important for transitioning between non-reactive and reactivated astrocytes 15, 49, 50. Similarly, astrocytic expression of SREBF2 the central transcription factor coordinating the expression of cholesterol biosynthesis genes as well as SLC1A2 a glutamate transporter were shown to be essential for neuronal function an viability 51-53. Repression of these genes in siHMG20A treated astrocytes was confirmed by QT-PCR (**Figure 3G**). Taken together these results indicate that HMG20A coordinates key genetic components governing astrocyte phenotypic state.

### *Hmg20a* repression in astrocytes blunts mitochondrial metabolism sensitizing cells to apoptosis

**Figure 4:**
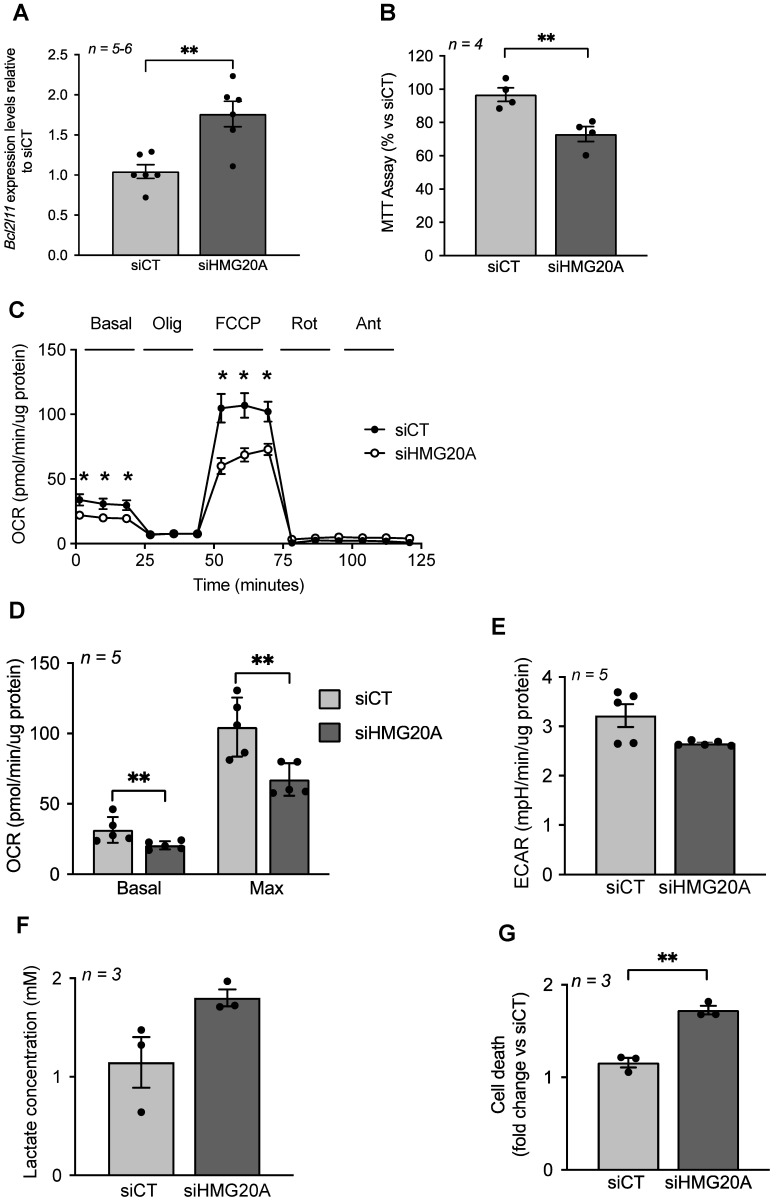
** HMG20A silencing alters mitochondrial bioenergetics sensitizing astrocytes to apoptosis.** (**A**) *Bcl2l11* transcript levels in siCT or siHMG20A treated astrocytes. Expression levels of *Bcl2l11* were normalized to the housekeeping gene *Gapdh. n = 5* independent experiments, ** *p < 0.01* unpaired Student *t-*test siCT versus siHMG20A. (**B**) Knockdown of HMG20A in astrocytes significantly decreased mitochondrial metabolism as assessed by MTT assay. Seahorse analysis showing (**C**) OCR profile plot, (**D**) Basal and maximal respiration and (**E**) ECAR in astrocytes silenced or not for HMG20A. Oligo; Oligomycin, FCCP; Carbonyl cyanide-p-trifluoromethoxyphenylhydrazone, Rot; Rotenone, Ant, Antimycin A. *n = 5* (independent primary cultures obtained from 4 male pups of 2-4 days old), * p < 0.05 unpaired *t-*test siCT versus siHMG20A. (**F**) Lactate secretion and (**G**) Apoptosis was assessed subsequent to siRNA-mediated HMG20A silencing in astrocytes. (**B**), (**F**) and (**G**) Data are referred to the average value detected in non-silenced astrocytes (siCT). For each condition, 3-4 biological replicates (independent primary cultures obtained from 4 male pups of 2-4 days old) were analyzed in duplicate. **p < 0.01 unpaired Student *t-*test siCT versus siHMG20A.

Although up-regulated genes did not cluster into functional hubs, individual genes such as *Bcl2l11* (*aka Bim*) were significantly increased in siHMG20A-treated cells. *Bcl2l11* is of particular interest in view of its outer mitochondrial membrane localization and its role in regulating mitochondrial bioenergetics as well as BAX-dependent cell apoptosis 54. As mitochondrial dysfunction, a hallmark of increased *Bcl2l11* expression, was recently found to impede reactive astrogliosis and to enhance neuronal cell death 55, we assessed mitochondrial metabolism in siHMG20A-repressed astrocytes. We first confirmed that *Bcl2l11* expression was increased in siHMG20A knockdown astrocytes (**Figure 4A**). These cells displayed a 30% reduction in NADH oxidation as assessed by the MTT assay suggestive of lower mitochondrial bioenergetics (**Figure 4B**). To further explore this premise, we measured oxygen consumption rate (OCR) and extracellular acidification rate (ECAR) in astrocytes silenced or not for *Hmg20a* using the seahorse XF-24 bioanalyzer. Basal and maximal respiration (following the addition of FCCP) were significantly reduced while proton leakage (following the addition of oligomycin) was unchanged in siHMG20A-transfected astrocytes as compared to siCT-treated cells (**Figure 4C and D**). The extracellular acidification rate (ECAR) was not altered consistent with sustained lactate secretion (**Figure 4E and F**). In line with lower ATP production, *Hmg20a* knockdown astrocytes were more susceptible to apoptosis (**Figure 4G**). Taken together these results indicate that HMG20A likely *via* BCL2L11 is required to sustain normal mitochondrial oxygen consumption and astrocyte survival.

### Hmg20a expression in astrocytes is mandatory for motoneuron survival *in vitro*

**Figure 5:**
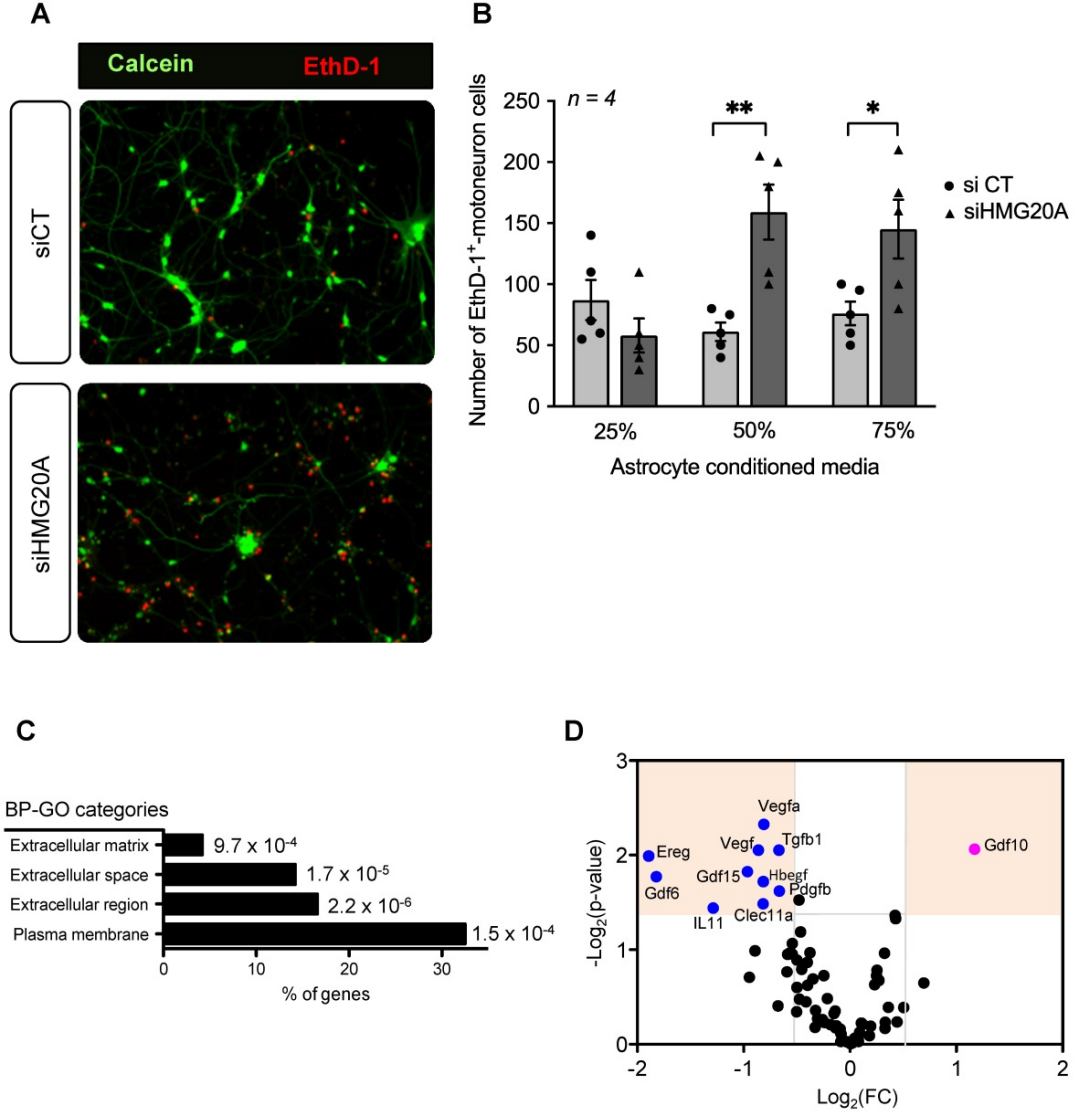
** HMG20A coordinates expression of astrocyte derived extracellular factors involved in neuronal survival.** (**A**) Representative immunofluorescent images of mouse spinal cord motoneurons cultured for 24 h in increasing percentage of conditioned media derived from primary astrocytes silenced for HMG20A (siHMG20A) or not (siCT). Live cells are stained with Calcein (green) while dying cells label for Eth-D1 (red). (**B**) Quantification of motoneuron cell death after growth in the indicated % of astrocyte conditioned media; control siCT astrocytes (black circles) or HMG20A silenced siHMG20A astrocytes (Black triangles). *n = 6* biological replicates (independent spinal cord motoneurons primary cultures). * p < 0.05 and ** p < 0.01, unpaired *t-*test siCT versus siHMG20A. (**C**) GO Cellular compartment of genes differentially regulated by siHMG20A in astrocytes. % of genes of the indicated categories is represented. *p-*value of the enrichment is also provided. (**D**) Vulcano plot showing expression change (logs(FC) and *p*-value (-log10(p-value)) (siHMG20a versus siCT) of all expressed growth factors encoding genes.

Our RNA-seq analysis pinpoint to a role of HMG20A in coordinating key molecular pathways transitioning astrocytes between a non-reactive and reactive phenotype, both mandatory for neuronal survival, pending environmental cues. As such, to functionally translate this molecular premise, isolated primary mouse motoneurons were cultured with increasing amounts of conditioned media derived from either siCT or siHMG20A-treated astrocytes and cell viability was assessed 24 h post incubation (**Figure 5A**). In cultures using conditioned media from HMG20A-silenced astrocytes, we observed a media mixture-dependent increase in motoneuron apoptosis reaching a maximum of 3-fold when using a 50% ratio (**Figure 5B**) validating the essentiality of *Hmg20a* expression in astrocytes to support motoneurons survival likely via extracellular factors. Accordingly, further analysis of our RNA-seq data revealed that several GO cellular compartments with genes related to the extracellular space were significantly altered in siHMG20A-treated astrocytes (**Figure 5C**). More specifically, we found *an armada* of growth factors some of which are key in neuron survival and function such as *Gdf15*, *Il11 and Tgfb1* to be significantly blunted in siHMG20A-treated astrocytes 56-58 (**Figure 5D**). Oddly, *Gdf10* was the only up-regulated growth factor in HMG20A silenced astrocytes. As HMG20A was detected in human serum, (**Figure S5**) we pondered whether astrocytes could potentially secrete the factor and impact neuronal survival. Secreted HMG20A concentrations in culture media were below detectable levels suggesting that astrocytes do not release this factor (data not shown). Taken together these results confirm that the molecular alterations induced by HMG20A depletion not only impacts astrocytes phenotypic polarization but also their capacity to convey survival signals to motoneurons *in vitro*.

### Pharmacological inhibition of the HMG20A-regulated LSD1-CoREST complex induces reactive astrogliosis and rescues hypothalamic neuron survival *in vitro*

**Figure 6:**
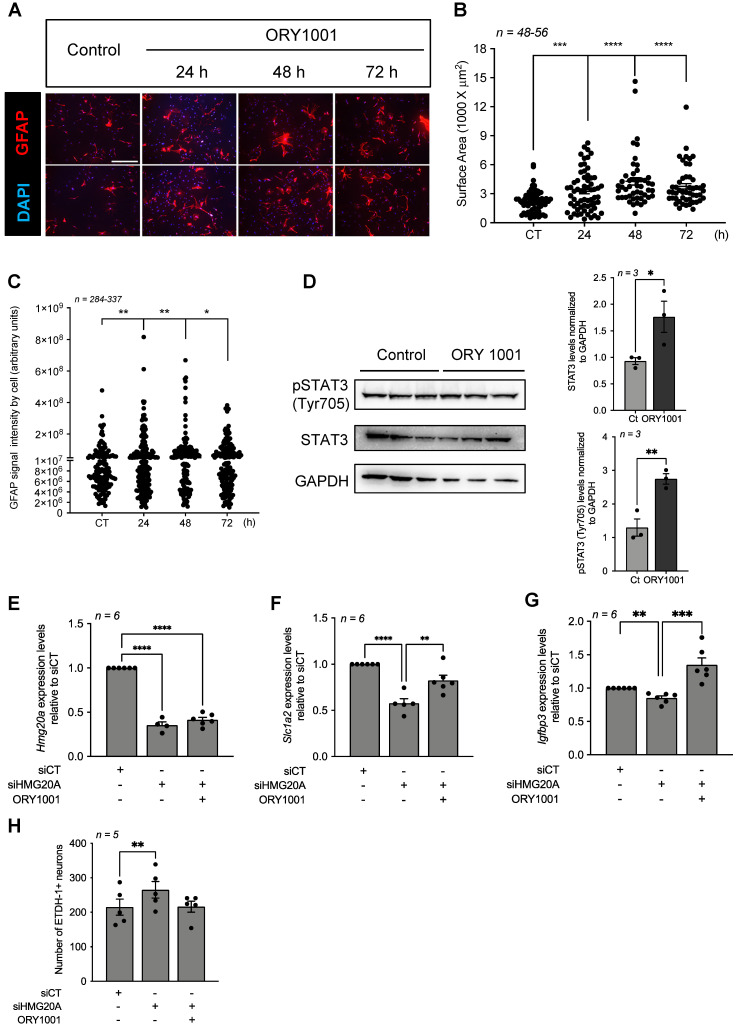
** The LSD1 inhibitor ORY1001 mimics HMG20A-mediated reactive astrogliosis and improves hypothalamic neurons survival.** (**A**) Representative immunofluorescent images of primary astrocytes treated or not for 24, 48 and 72 h with ORY1001. Cells were immunostained for GFAP (red) and nuclei with DAPI (blue). Two images are provided for each time point. Scale bar: 500 um. (**B**) Quantification of reactive astrocytes (i.e. reactive astrogliosis) in (**A**) as assessed by GFAP expression and average cell surface area. *n = 46-56* dot representing an image from which the area of at least 40 GFAP^+^ cells were measured from 3 independent slides/experiments. (**C**) Quantification of reactive astrocytes in (**A**) as assessed by GFAP intensity. *n=337-396* cells from 3 independent slides/experiments. **** *p < 0.0001*, *** *p < 0.001*, ** *p < 0.01* and *p < 0.05 unpaired two-tailed *t-*test CT versus 48 and 72 h. (**D**) Protein levels (left panel) and quantification (right panels) of STAT3 and phosphorylated STAT3 (pSTAT3) were assessed in whole cell extracts of astrocytes cultured or not with ORY1001 for 72 h. Relative expression levels were normalized to GADPH. Each lane represents an independent experiment. Transcript levels of (**E**) *Hmg20a* (**F**) *Slc1a2* and (**G**) *Igfbp3* were determined in astrocytes silenced or not for HMG20A and treated or not with ORY1001. *n = 6* independent experiments. **** *p < 0.0001*, *** *p < 0.001* and ** *p < 0.01* unpaired two-tailed *t-*test siCT versus siHMG20A and siHMG20A/ORY1001. (**H**) Quantification of primary hypothalamic neuron cell death after growth in media containing 75 % of astrocyte conditioned media obtained from siCT-treated astrocytes, siHMG20A-treated astrocytes and ORY1001 supplemented siHMG20A-treated astrocytes. *n = 5* biological replicates (independent hypothalamic neuron primary cultures). ** *p < 0.01*, ANOVA, Turkey's multiple comparison test.

A key molecular regulatory function of HMG20A is to counteract HMG20B-mediated activation of the LSD1-CoREST complex that facilitates demethylation of H3K4me1/2 via LSD1 (*aka* KDM1A) resulting in the repression of neuron-specific genes 26. More recently, we demonstrated that inhibition of HMG20A blunted EMT, a process also implicating LSD1 31. Since reactive astrogliosis was defined as an EMT-like process 15, we posit that pharmacologically mimicking the action of HMG20A in repressing the LSD1-CoREST complex should induce reactive astrogliosis. Towards addressing this premise, primary astrocytes were treated with ORY1001, a potent and selective covalent inhibitor of LSD1 59. ORY1001 treated GFAP^+^-astrocytes displayed time-dependent hypertrophy with increasing cellular processes, doubling their surface area at 48-72 h post-treatment as compared to untreated cells collected at 72 h post-treatment (**Figure 6A and B**). Upregulation of GFAP as well as VIMENTIN has been associated with reactive astrogliosis in several, but not all, CNS diseases 12. Herein, although we did not detect any differences in levels of both proteins by Western blot analysis (**Figure S9**), quantification of GFAP immunostaining signal intensity at the single cell level revealed a significant time-dependent increase in ORY1001 treated astrocytes as compared to untreated cells (**Figure 6C**). To further substantiate these immunostaining results we assessed STAT3 phosphorylation levels that were shown to be stimulated during reactive astrogliosis under widespread stress conditions 11. Accordingly, both STAT3 and pSTAT3 were increased in astrocytes treated with ORY1001 for 72 h (**Figure 6D**). These data suggest that both HMG20A and ORY1001 induce reactive astrogliosis via the inhibition of the LSD1-CoREST complex. We next reasoned that treatment of HMG20A-depleted astrocytes, in which the LSD1-CoREST complex is active, with ORY1001 should rescue the expression of genes implicated in reactive astrogliosis. Such premise was confirmed by demonstrating the recovery in the expression levels of both *Slc1a2* and *Igfbp3*, but not of *Hmg20a* (see **Figure 3**) in ORY1001-treated HMG20A ablated astrocytes to levels similar or higher to those of siCT-astrocytes (**Figure 6E-G**). Furthermore, hypothalamic neuron cell death induced by condition media obtained from siHMG20A-depleted astrocytes was negated by ORY1001 treatment (**Figure 6H**). We thus conclude that mechanistically ORY1001 as well as HMG20A repress the LSD1-CoREST complex either through HMG20B or LSD1 thereby alleviating a molecular/epigenetic break that restrains activation of the reactive astrogliosis genetic program that includes *Slc1a2* and *Igfbp3*.

### ORY1001 treatment improves high-fat high-sucrose-induced glucose intolerance in mice with a concomitant decrease in hypothalamic HMG20A expression

**Figure 7:**
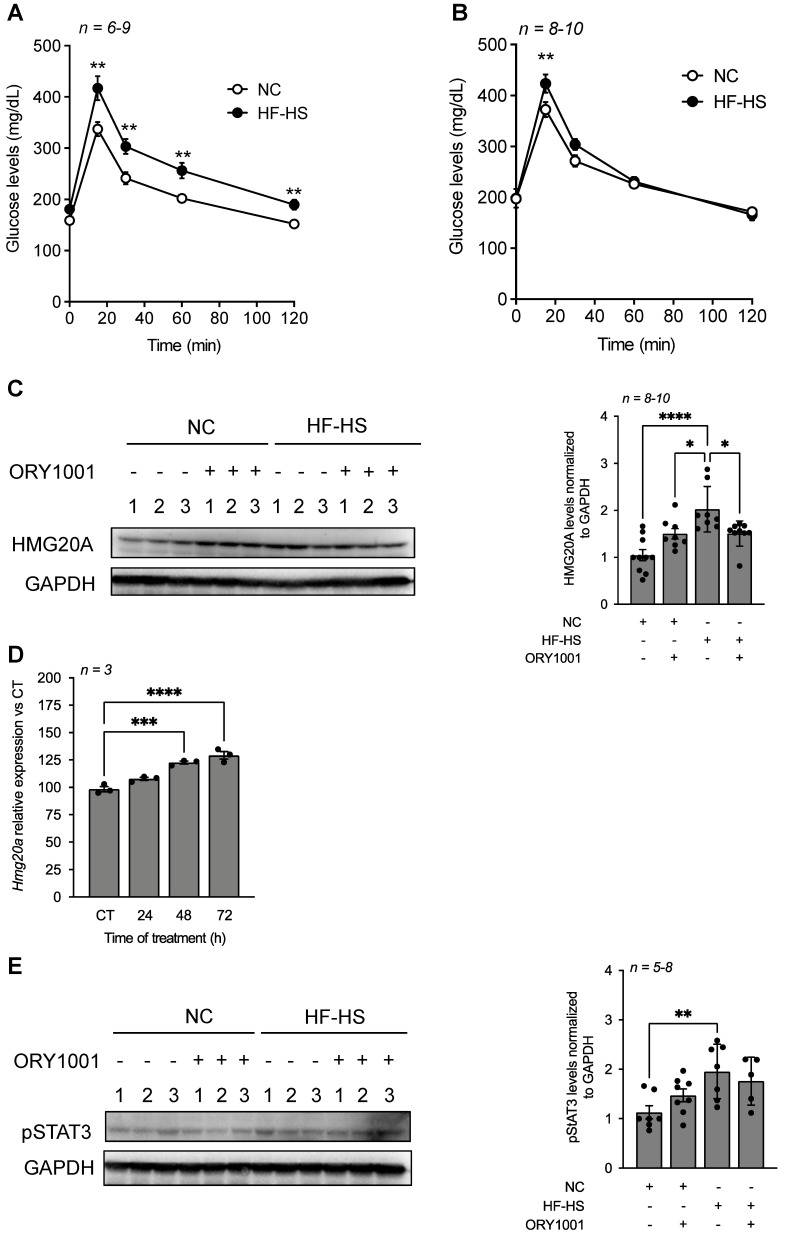
** ORY1001 improves glucose sensitivity in HF-HS fed mice correlating with decreased hypothalamic HMG20A levels.** An oral glucose tolerance test was performed on either normal chow (NC) or high-fat high-sucrose (HF-HS) fed mice for 6 weeks that were either (**A**) untreated or (**B**) treated with ORY1001 (0.0125 mg/Kg body weight). Inset depict area under the curve (AUC). ****p < 0.0001, **p < 0.01 and *p < 0.05 unpaired *t*-test CT versus HF-HS. (**C**) Representative Western blot depicting HMG20A protein levels in hypothalamus extracted from 3 mice for each condition depicted in (**A**) and (**B**). Right panels depict the quantification of blots for *n = 3-10*. GAPDH was used as the housekeeping protein for normalization. **** *p< 0.0001* and *p < 0.05, unpaired *t*-test CT versus HFD. (**D**) Relative expression levels of *Hmg20a* in isolated primary astrocytes treated with ORY1001 for up to 72 h. Transcript levels were normalized to those of the housekeeping gene *Gapdh*. *n = 3* independent experiments. ***p < 0.001, ****p < *0.0001*, unpaired *t*-test CT versus experimental time of OR1001 treatment. (**E**) Representative Western blot depicting pSTAT3 protein levels in hypothalamus extracted from 3 mice for each condition depicted in (**A**) and (**B**). Right panels depict the quantification of blots for *n = 5-8*. GAPDH was used as the housekeeping protein for normalization. **p < 0.01, unpaired *t*-test CT versus HF-HS.

To assess whether the *in vitro* beneficial effects of ORY1001 could be translated *in vivo* by protecting against high fat-induced neuroinflammation and insulin resistance, mice were exposed to a high-fat high-sucrose (HF-HS) diet for 6 weeks and treated or not with ORY1001. HF-HS fed mice exhibited severe glucose intolerance as compared to normal chow (NC) fed mice within 6 weeks of regimen. Of note, blood glucose levels in HF-HS fed mice remained significantly higher at 120 min post glucose bolus as compared to normal chow fed mice (**Figure 7A**). In contrast, ORY1001-treated HF-HS fed mice displayed normal glucose tolerance as compared to NC counterparts (**Figure 7B**). Notwithstanding, basal blood glucose levels were slightly higher, but not significantly, in the ORY1001-treated group independent of regimen (**Figure 7A and B**). Interestingly, assessment of HMG20A expression in the hypothalamus of these mice, revealed a slight increased, albeit nonsignificant, in protein levels in ORY1001-treated NC fed mice suggesting a potential direct regulation of the compound on HMG20A expression (**Figure 7C**). This was confirmed by demonstrating that *Hmg20a* transcript levels were time dependently increased in ORY1001-treated primary astrocytes (**Figure 7D**). Consistent with our aforementioned results (**Figure 2C**), HMG20A protein levels were significantly increased in the hypothalamus of HF-HS mice as compared to NC mice. Remarkably, ORY1001 treatment repressed HMG20A levels to those detected in ORY1001-treated NC fed mice (**Figure 7C**). A similar expression pattern was observed for pSTAT3, albeit the decrease detected in ORY1001-treated HF-HS mice was not significant when compared to HF-HS fed animals (**Figure 7E**) Taken together these results suggest that ORY1001 treatment improves glucose tolerance with a corresponding resorption of reactive astrogliosis likely as a result of reduced neuroinflammation.

## Discussion

The overall aim of the current study was to extend our recent findings, demonstrating that the metabesity-linked chromatin factor HMG20A coordinates pancreatic islet beta-cell maturation and functional output in response to altered glucose levels 17, to astrocytes, the major glucose-metabolising cell subpopulation within the brain hypothalamus. We find that: (1) HMG20A is highly expressed in astrocytes within the arcuate and ventromedial nuclei of the hypothalamus cells, the nexus of brain-regulated glucose homeostasis, (2) NeuN^+^ neurons, but not oligodendrocytes or microglia, also express HMG20A, (3) HMG20A levels are increased in hypothalamic astrocytes of pre-diabetic and obese mice, and in line with its involvement in tissue adaptive response, correlating with higher levels in WAT samples of obese non-diabetic individuals, (4) *HMG20A* levels collapse in obese diabetic patients as compared to obese non-diabetic individuals, (5) HMG20A coordinates expression of strategic hubs involved in astrocyte reactivity, (6) HMG20A depletion impairs astrocyte function and neuron survival and (7) pharmacological inhibition of the LSD1-CoREST complex, mimicking HMG20A mechanism of action, induces reactive astrogliosis. Therefore, we conclude that *HMG20A* is a key molecular regulator of reactive astrogliosis fine-tuning the reactivity degree in response to physiological cues with the ultimate purpose to protect and/or control damage of neurons. In contrast, the functional impact of HMG20A in NeuN+ neurons remains to be determined as no current data is available apart from its role in neuronal development 26.

Our results indicate that in the context of metabesity neither glucose nor lipids individually are influencers contributing to enhanced *Hmg20a* expression in astrocytes and their subsequent reactive phenotype. Although counterintuitive, in view of astrocytes intimate contact with the blood brain barrier (BBB) and function as energy sensor, our results corroborate previous studies demonstrating that under physiological conditions reactive astrogliosis does not occur in isolation, but as part of a coordinated and multicellular response to CNS insult that includes a crosstalk with neurons and microglia 16. As such, consumption of a HFD was shown to induce expression of IL1B, and IL6 as well as TNFA in mouse neurons as well as to convey a pro-inflammatory phenotype to microglia within the hypothalamic arcuate nucleus in both mice and humans 6, 60, 61. Such cytokines have been recognized as paracrine instructive signals promoting reactive astrogliosis and thus could be mediating the HFD-induced HMG20A expression *in vivo* 49. This is consistent with the knowledge that reactive astrocytes are found at site of CNS injury likely induced by the initial pro-inflammatory environment triggered by damaged neurons and activated microglia 12.

As discussed above, reactive astrogliosis is an inflammatory process that initially conveys neuron protection but that long-term needs to be resorb to avoid detrimental damage. Interestingly, a negative feedback loop between the LSD1-CoREST complex and the orphan nuclear receptor NURR1 was shown to attenuate simultaneously the pro-inflammatory response in astrocytes and microglia. The authors concluded that such crosstalk blunts a feed forward amplification of the NF-kB mediated inflammatory response restoring a non-reactive phenotype to both microglia and astrocytes thereby protecting dopaminergic neurons from inflammation-induced cell death 62. These findings are contextually relevant as a key regulatory function of HMG20A is also to counteract the effects of the LSD1-CoREST complex pinpointing to a strategic function of HMG20A in potentiating astrocyte reactivity. This premise was validated at the cellular level by demonstrating that the potent inhibitor of the LSD1-CoREST complex, ORY1001 induced *Hmg20a* expression in astrocytes and reactive astrogliosis. At the molecular level, transcriptome profiling revealed the regulatory function of HMG20A on key genetic hubs, including the inflammatory response involved in reactive astrogliosis. Of particular relevance for the transitory status of reactive astrogliosis, numerous genes within the inflammatory hub that were down regulated by siHMG20A appear to encode anti-inflammatory and pro-regenerative factors (**Figure 3D**). For instance, TLR3 was shown to be induced in human astrocytes upon inflammation and when activated, to convey a neuroprotective response rather than a pro-inflammatory reaction 63. In addition, although chronic high levels of CCL5 have been associated with MS 64, this chemokine was reported to promote an anti-inflammatory resolution-phase of macrophage reprogramming under some settings 65. Likewise, IRF7 was shown to induce a pro-to-anti-inflammatory phenotypic shift in microglia 66. Finally, LIF through its immunomodulatory and regenerative properties promotes survival of neurons and glia in several animal models of neurodegenerative disease, such as amyotrophic lateral sclerosis, MS, spinal cord injury and stroke 67. These data suggest that HMG20A modulates reactive astrogliosis, both at its initiation as well as at its reabsorption. It is therefore tempting to speculate that the differentiation/polarization status/level of astrocytes is a continuum of changes which is dictated by the interaction of HMG20A and/or NURR1 with the LSD1-CoREST complex favoring an anti-inflammatory state of reactive astrocytes with pro-survival and pro-regenerative properties. Similarly, in our previous studies in islet beta cells, we detected a transitory increase in HMG20A levels during pregnancy, correlating with the adaptation phase that takes place in islets in response to increase insulin demands 17, 18. As such, under environmental insults such as hyperglycemia, HMG20A could activate on one hand the adaptation pathways conveying maturation/functionality of beta cells in islets and on the other hand transitory reactive astrogliosis and neuronal protection in CNS. This molecular model could rationalize our findings that obese non-diabetic individuals have higher levels of HMG20A in WAT favoring an anti-inflammatory and pro-survival cell phenotype whereas obese diabetic patients expressed normal levels of HMG20A indicative of a non-reactive cell phenotype incapable of relaying cell protection and repair ultimately leading to neurodegeneration disease such as MS or AD as well as other complications such as altered glucose homeostasis. In this context it is also of interest, that expression of the glucose transporter *Slc2a1* (GLUT1) was inhibited in HMG20A-silenced astrocytes. Garcia-Caceres and colleagues recently reported that GLUT1 levels were decreased in astrocytes-specific ablation of the insulin receptor coupling insulin signaling to brain glucose uptake and nutrient availability 9. As alterations in the insulin-CNS axis have been associated with AD 8, HMG20A may contribute to disease development via restriction of insulin/GLUT1-induced glucose flux through astrocytes. This model will require further validation including the challenging task of assessing HMG20A expression levels in hypothalamus of obese non-diabetic and obese diabetic individuals.

Our molecular analysis also revealed that silencing of HMG20A repressed the EMT signaling program including the triggering factor TGF-beta. These results substantiate our previous study in which we demonstrated that HMG20A together with the LSD1-CoREST complex is required for TGF-beta-mediated EMT in mouse mammary epithelial NMuMG cells 31. Reactive astrogliosis was recently defined as an EMT-like process that fine tunes astrocyte reactivity in response to injury and tissue repair 15. As such a parallel between reactive astrogliosis and wound healing was proposed including a role of astrocytes in trans-differentiating into functional neurons, a process facilitated by VEGF 68, 69. This growth factor along with a subset of genes implicated in cytoskeleton-integrin connection previously shown to be involved in wound healing were down regulated in siHMG20A treated astrocytes. Taken together these molecular data further support our model that HMG20A likely potentiates the degree of reactive astrogliosis promoting an anti-inflammatory and pro-regenerative phenotype, analogous to our recent studies establishing the role of the orphan nuclear receptor LRH-1/NR5A2 in immunomodulation and trans-regeneration of islet beta cells 42, 70, 71.

We also provide functional data derived from our molecular analysis highlighting a cell autonomous role of HMG20A in promoting astrocyte viability, activity and differentiated/reactive state. We show that HMG20A is required to sustain normal levels of mitochondrial oxygen consumption since its deficiency leads to impaired bioenergetics emphasized by reduced basal and maximum oxygen consumption, which might contribute to sensitive astrocytes to apoptosis. Of particular interest, mitochondrial dysfunction was shown to impede the generation of reactive astrocytes fostering neuronal cell death consistent with the finding that reduced reactive oxygen species (ROS) in astrocytes induced major alterations in brain energy and redox metabolism leading to impaired neuronal function and cognitive learning 55, 72. These studies are in line with our findings that HMG20A through various cellular pathways including mitochondrial metabolism coordinates astrocyte phenotypic state and physiological activity. We argue that these functional consequences are likely, in part, conveyed by HMG20A-regulated BIM expression. Indeed, in addition to its known role in apoptosis, BIM was also shown to regulate mitochondrial activity. Loss of BIM was found to increase oxygen consumption characterized by higher basal and maximum respiration rates in mouse embryonic fibroblast 54 corroborating our findings that increased expression results in decreased mitochondrial bioenergetics. These results are in line with a previous study revealing that BIM potentiates mitochondrial membrane potential 73.

In parallel, the capacity of HMG20A to potentiate reactive astrogliosis through the LSD1-CoREST complex was functionally validated using the LSD1 inhibitor ORY1001. Of high relevance, Vafidemstat (ORY2001), an oral small molecule that has been optimized for CNS indications and that acts as a covalent inhibitor of LSD1, similar to ORY1001, was shown to reduce neuroinflammation and improve cognitive behavior in animal models of AD and MS 74, 75 as well as to improve inflammation biomarkers in AD patients enrolled in a phase IIa clinical trial 76 (https://clinicaltrials.gov/ct2/show/study/NCT03867253). In view of the link between AD and T2DM 77, 78, these results, clearly suggest that targeting either HMG20A or the LSD1-CoREST complex in astrocytes may improve neuroinflammation and glucose homeostasis while salvaging pancreatic islet function in T2DM patients 2. In this framework, we provided proof-of-concept of such action by demonstrating that ORY1001 treatment improved glucose tolerance with a concomitant reduction in HMG20A expression levels, and to a lesser extent pSTAT3, in mice fed a HF-HS diet. These results also corroborate the model that resorption of neuroinflammation-induced reactive astrogliosis is likely essential to protect neurons against apoptosis. At the molecular level ORY1001 reversed the effect of HMG20A silencing on the expression of key beta-cell genes involved in insulin secretion 79 as well as reactive astrogliosis linked genes in astrocytes.

We also present functional data for a non-cell autonomous role of HMG20A in supporting neuron survival. Accordingly, a great number of secreted growth factors, previously shown to promote neuron survival and function 12 were down-regulated in HMG20A knockdown astrocytes. The latter suggests that astrocytes harbor a repertoire of secreted factors capable of jointly supporting neuron survival and regeneration as exemplified by the recent finding that transplanted immature astrocytes do not polarize to a pro-inflammatory reactive phenotype after CNS injury, but rather, favor neuron outgrowth and improve cell therapeutic outcomes in a Parkinson's disease mouse model 80, 81. Notwithstanding, a single growth factor, GDF10 *aka* bone morphogenetic protein 3 (BMP3), was significantly up-regulated while other members such as GDF6/BMP13 and GDF15/placental BMP were down-regulated in astrocytes lacking HMG20A. BMPs form a subgroup in the TGF-beta superfamily and act as powerful morphogens that play dynamic roles in the development of the brain early on in life, during which they sequentially induce neurogenesis and then astrogliogenesis 82. In adult, both GDF6/BPM13 and GDF15/placenta BMP were found to possess potent trophic effect on motor and sensory neurons 56, 83. Of particular importance was the finding that BPM6 conveys a protective effect against amyloid beta-induced neurotoxicity, a hallmark of AD, in rat hippocampal neurons 84 Interestingly, GDF10/BMP3 is a unique member of this family as it functions as an antagonist of the BMP pathway 85. As such the opposing regulation of GDF6 and GDF15 as compared to GDF10 emphasises again the role of HMG20A in establishing a pro-survival and tissue repair molecular phenotype to astrocytes. Intriguingly, lactate secretion was also increased, albeit not significantly, that should have resulted in neuroprotection, as reported in several CNS insults 86. Yet, both motoneurons and hypothalamic neurons survival was decreased in conditioned media from siHMG20A-silenced astrocytes. In this sense, the gene encoding the glutamate transporter SLC1A2 (*aka* EAAT2) implicated in the uptake and recycling of glutamate released from neurons thereby maintaining extracellular levels low and avoiding excitotoxicity was significantly decreased in siHMG20A silenced astrocytes 87. As such, reduction in glutamate removal from the extracellular space leading to toxic levels may counteract the beneficial effects of lactate on neuronal survival.

**Figure 8:**
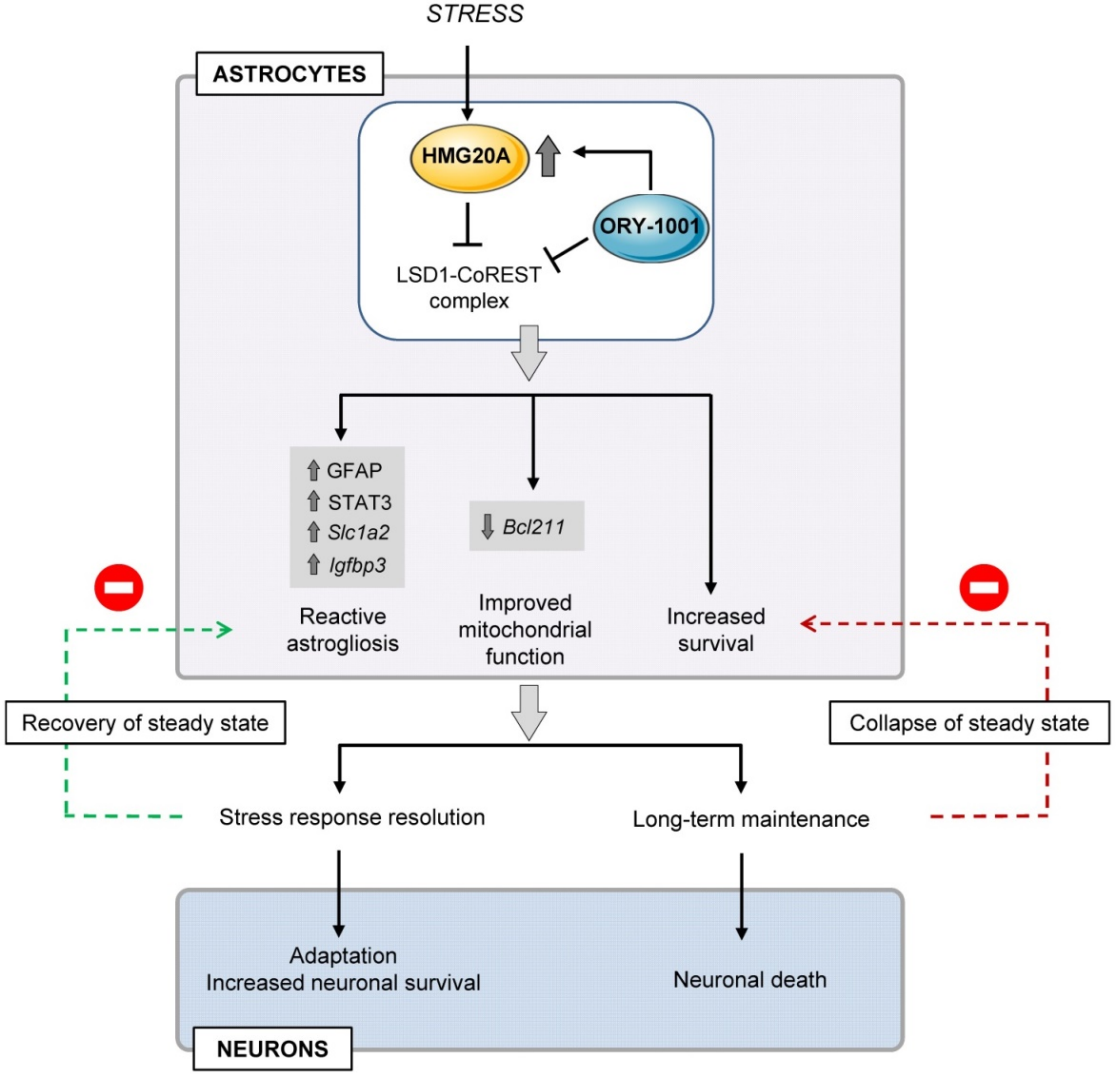
** Proposed model for HMG20A/ORY1001 mechanism of action**. Under stress conditions, HMG20A expression is enhanced in hypothalamic astrocytes resulting in the inhibition of the LSD1-CoREST complex and downstream activation of the genetic program for reactive astrogliosis, enhanced mitochondrial function and increased cell survival. This phenotypic switch will promote neuronal protection and survival with the goal to resolve the stress response, re-establish a physiological steady state and resorption of reactive astrogliosis. If unresolved, sustained reactive astrogliosis will precipitate both astrocyte and neuron dismay and apoptosis. Treatment of astrocytes with the LSD1 inhibitor ORY1001 mimics HMG20A action, opening the possibility of its pharmacologic use for neuronal protection in metabesity conditions.

The overall data presented herein allocate a molecular and functional link between HMG20A and astrocyte reactivity. HMG20A regulates molecular hubs modulating phenotypic changes needed for astrocytes to respond to different physiological situations such as metabesity in order to prevent CNS collapse (**Figure 8**). Failure of those HMG20A-mediated processes leads to astrocyte dysfunction and ineptitude to react resulting in neuron death and to the potential development of neurodegenerative diseases such as AD as well as T2DM, for which a link has previously been established. As consequence, our study opens a venue to consider targeting HMG20A regulatory function, as exemplified by ORY1001, to treat such diseases.

## Supplementary Material

Supplementary figures and tables.

## Figures and Tables

**Table 1:** Clinical characteristics of Pizzara subjects who donated blood samples.

	Non-obese/non-diabetic	Obese/non-diabetic	Obese/diabetic	Non-obese/diabetic
n	18	9	13	12
Age (years)	48.1±5.3	49.3±7.0	56.1±6.8	52.9±8.9
BMI (kg/m^2^)	26.1±2,8	34.1±3.4	36,1±4.1	27.4±2.4
Waist circumference (cm)	88.7±7.9	103.6±6.1	110.6±9.3	94.3±6.9
Waist to hip ratio	0.86±0.06	0.91±0.08	0.99±0.08	0.96±0.08
Systolic blood pressure (mmHg)	124.6±16.6	116.2±7.9	144.7±17.6	129.9±12.5
Diastolic blood pressure (mmHg)	70.7±8.3	72.8±3.9	82.2±11.0	77.0±9.7
Fasting Glucose (mmol/L)	5.4±0.4	5.5±0.4	7.4±0.7	7.1±0.6
Glucose post OGTT (mmol/L)	6.2±0.9	6.5±0.9	9.9±2.4	9.8±3.4
HbA1c (%)	5.4±0.3	5.5±0.2	6.2±0.5	5.9±0.7
Fe (micromol/L)	14.8±5.8	15.0±5.1	13.3±4.5	17.8±7.7
Adiponectin (mg/L)	22.9±9.2	22.4±13.5	12.8±12.9	13.8±8.4
Δglycemia	1.43±7.18	-0.92±9.85	2.81±8.24	4.67±9.71
ΔHbA1c	0.81±5.62	4.16±3.60	2.19±5.98	-1.01±10.08

**Table 2:** Clinical characteristics of subjects whom underwent an adipose tissue biopsy.

	Non-obese/non-diabetic	Obese/non-diabetic	Obese/diabetic	Non-obese/diabetic
n	8	8	8	3
Age (years)	47.7±9.2	40.8±8.6	49.0±6.9	51.3±8.9
BMI (Kg/m2)	26.7±2.8	53.4±7.7	53.9±8.6	25.3±3.5
Waist (cm)	94.4±5.2	139.8±20.0	144.2±14.0	107.3±2.5
Waist to Hip ratio	0.93±0.02	0.91±0.06	0.94±0.11	1.12±0.08
Systolic blood pressure (mmHg)	123.0±10.4	120.3±15.3	144.6±21.5	127.5±28.9
Diastolic blood pressure (mmHg)	80.6±6.8	84.0±5.2	88.6±10.2	71.5±4.9
Fasting Glucose (mmol/L)	4.86±0.64	5.25±0.42	7.78±3.23	9.17±2.86
HbA1c (%)	5.6±0.4	5.5±0.2	7.2±1.3	7.5±1.2
Fe (μmol/L)	17.5±9.8	11.9±3.7	10.9±4.5	13.8±2.3
Adiponectin (mg/dl)	15.2±6.5	18.9±8.2	10.8±8.2	8.4±0.9
