# Supplementary material for: The metabesity factor HMG20A potentiates astrocyte survival and reactive astrogliosis preserving neuronal integrity
Source: Theranostics. 2021 May 12;11(14):6983–7004. doi: 10.7150/thno.57237 (PMC8171100; doi:10.7150/thno.57237)
Supplement: Supplementary file 1 — Supplementary figures and tables. [file thnov11p6983s1.pdf]

Supplemental Material

The metabesity factor HMG20A potentiates astrocyte survival and reactive astrogliosis preserving neuronal integrity

Petra I. Lorenzo *et al.*

ID	Forward primer	Reverse primer
**Mouse**		
Hmg20a	AACCAACCCAGAGTTTGTGG	TTGCTCATCTTCAGGCCTTT
Gfap	GGGGCAAAAGCACCAAAGAAG	GGGACAACTTGTATTGTGAGCC
Vimentin	CGTCCACACGCACCTACAG	GGGGGATGAGGAATAGAGGCT
Il1b	AACTGTTGGTGAGGAATGTGG	GGTCCTGTCCCTCTTGTTTTCA
Slc1a2	ACAATATGCCCAAGCAGGTAGA	CTTTGGCTCATCGGAGCTGA
Ereg	CTGCCTCTTGGGTCTTGACG	GCGGTACAGTTATCCTCGGATTC
Srebf2	GCAGCAACGGGACCATTCT	CCCCATGACTAAGTCCTTCAACT
Igfbp3	GACGACGTACATTGCCTCAG	GTCTTTTGTGCAAAATAAGGCATA
Tgfb1	CTCCCGTGGCTTCTAGTGC	GCCTTAGTTTGGACAGGATCTG
Vcam1	TTGGGAGCCTCAACGGTACT	GCAATCGTTTTGTATTCAGGGGA
Gapdh	CACCAACTGCTTAGCCCC	TCTTCTGGGTGGCAGTGATG
Cyclophilin	ATGGCAAATGCTGGACCAA	GCCATCCAGCCATTCAGTCT
**Human**		
HMG20A	GCATGAAGATGAGCAACGAA	GCTCATTCATGAACCGAACA
ACTB	AAACTGGAACGGTGAAGGTG	GTGGCTTTTAGGATGGCAAG
HIST1H2AB	CGGTGCTTGAGTACCTGACC	TTCACTTTCCCTTGGCCTTA

TABLE S1: List of primers used in this study.

Primary antibodies	Host	Dilution	Supplier	Catalog number
HMG20A	Rabbit	1:1000	Sigma-Aldrich	HPA008126
Insulin	Mouse	1:500	Sigma-Aldrich	I2018
Glucagon	Mouse	1:200	Sigma-Aldrich	A944
Somatostatin	Goat	1:100	Santa Cruz Biotechnology	SC-7819
Vimentin	Rat	1:200	Santa Cruz Biotechnology	SC-32322
GFAP	Mouse	1:500	Sigma-Aldrich	G3893
GAPDH	Rabbit	1:1000	Cell Signaling Technology	14C10
STAT3	Rabbit	1:1000	Cell Signaling Technology	9132
Phospho-STAT3	Rabbit	1:1000	Cell Signaling Technology	9145
IBA1	Rabbit	1:200	WAKO	019-19741
OLIG2	Mouse	1:100	Sigma-Aldrich	MABN50
NeuN	Mouse	1:100	Merk Millipore	M377
**Secondary antibodies**	**Host**	**Dilution**	**Supplier**	**Catalog number**
Rhodamine Red Anti-rabbit	Goat	1:400	Jackson ImmunoResearch	111-295-003
Fluorescein anti-mouse	Goat	1:400	Jackson ImmunoResearch	115-095-071
Alexa fluor 568 goat anti-rabbit	Goat	1:800	Thermo Fisher Scientific	A11011
Alexa fluor 488 goat anti-mouse	Goat	1:800	Thermo Fisher Scientific	A11001
HRP anti-rabbit	Goat	1:1000	Sigma-Aldrich	AP307P

TABLE S2: List of antibodies used in this study.

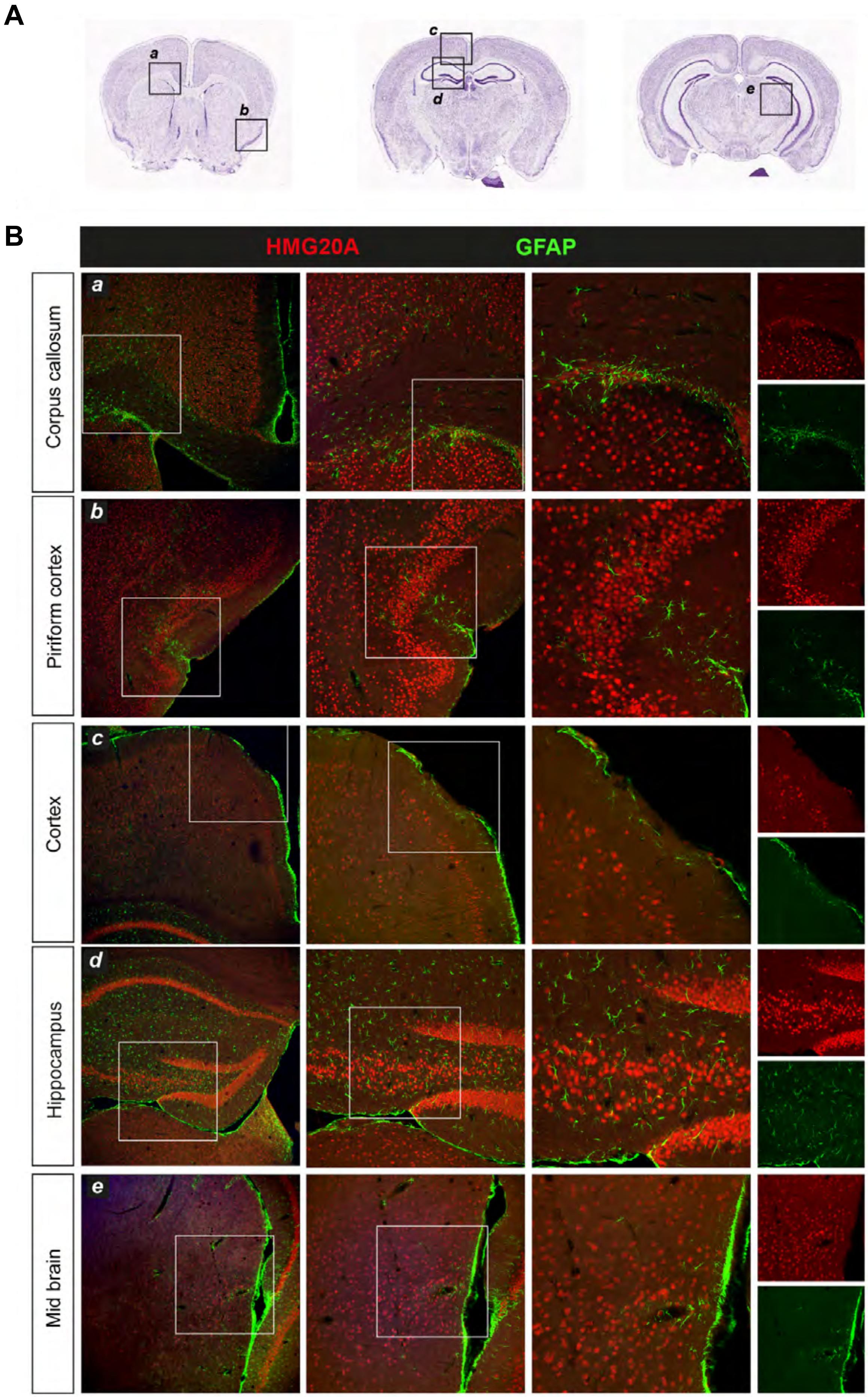

Figure S1. HMG20A expression pattern in mouse brain neurons. (**A**) Anatomical areas of the brain shown in (**B**) taken from the Allen Brain Atlas (https://mouse.brain-map.org/). (**B**)HMG20A (red) expression was assessed by immunofluorescence and was co-localized with NeuN (green, right panels) expressing neurons in various brain areas as depicted in the figure. Magnification: 10X

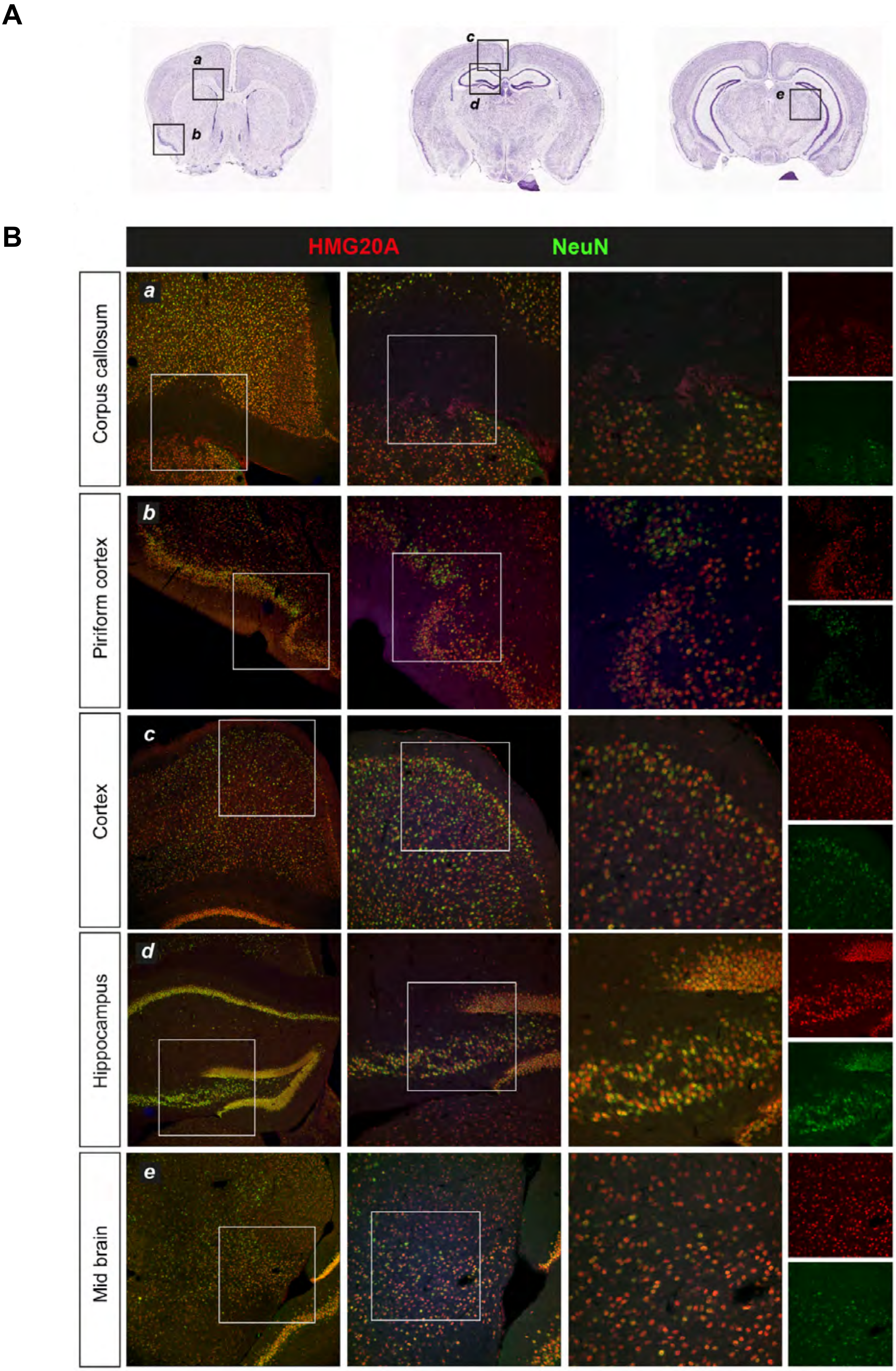

Figure S2. HMG20A expression pattern in mouse brain astrocytes. (**A**) Anatomical areas of the brain shown in (**B**) taken from the Allen Brain Atlas (https://mouse.brain-map.org/). (**B**) HMG20A (red) expression was assessed by immunofluorescence and was co-localized with NeuN (green, left panels) expressing astrocytes in various brain areas as depicted in the figure. Magnification: 10X

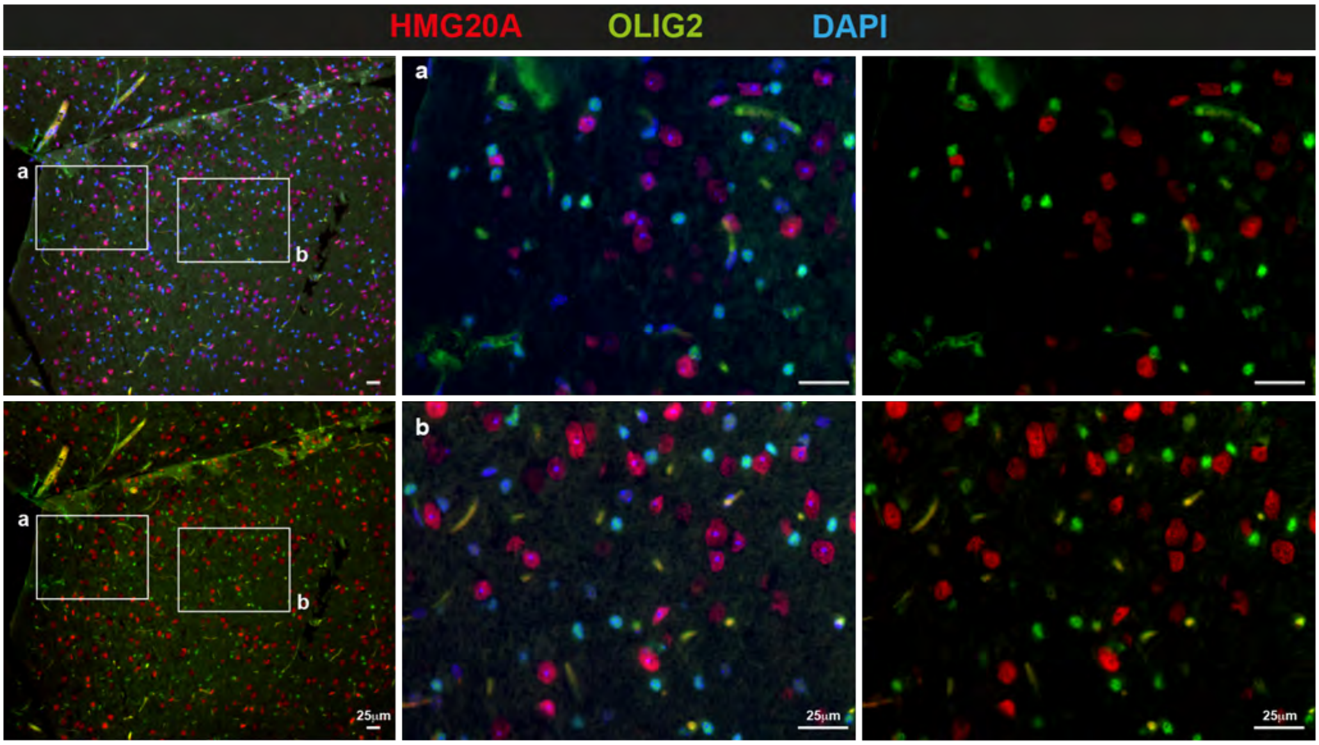

Figure S3. HMG20A is not expressed in oligodendrocytes. Fluorescence microscope images of brain paraffin sections immunostained for HMG20A (red) and the oligodentrocyte marker OLIG2. Scale bar: 25µm.

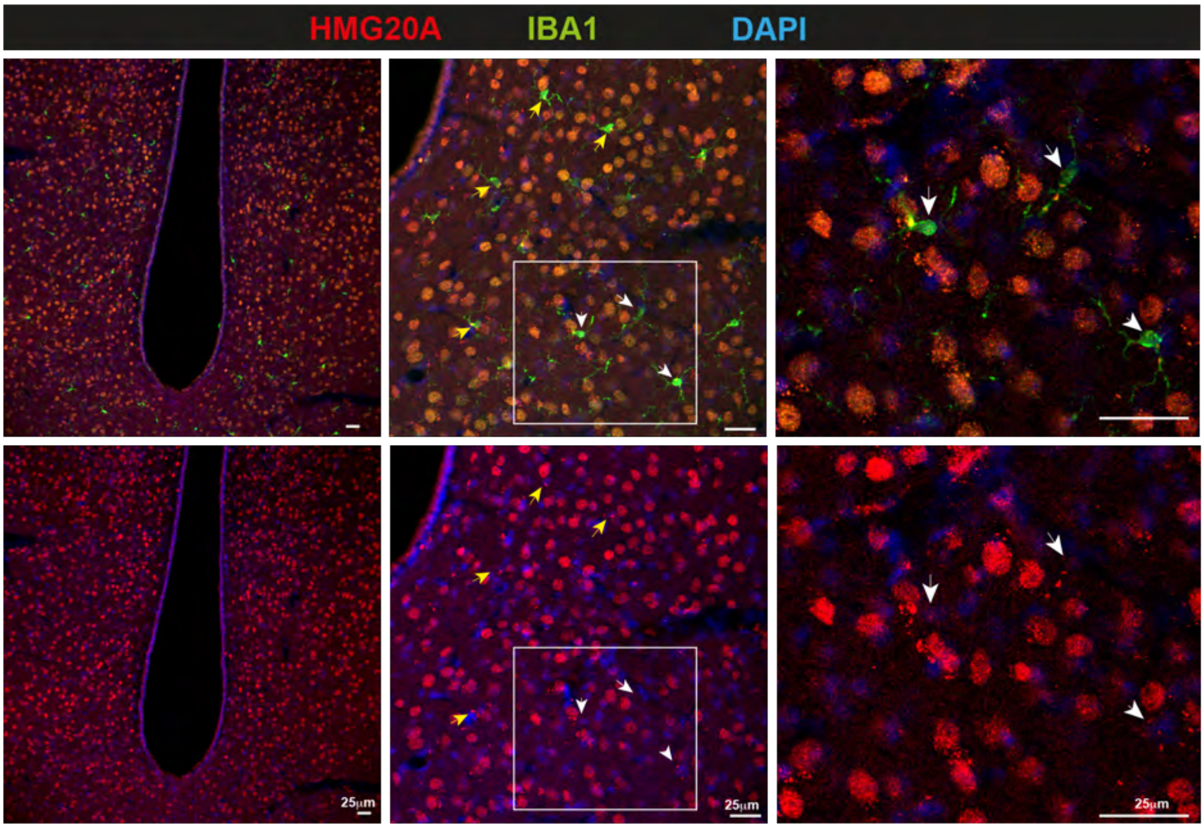

Figure S4. **HMG20A is not expressed in microglia**. Confocal microscope images of brain vibratome sections immunostained for HMG20A (red) and the microglia marker IBA1 (Green). Arrows point to microglial cells. Scale bar: 25µm.

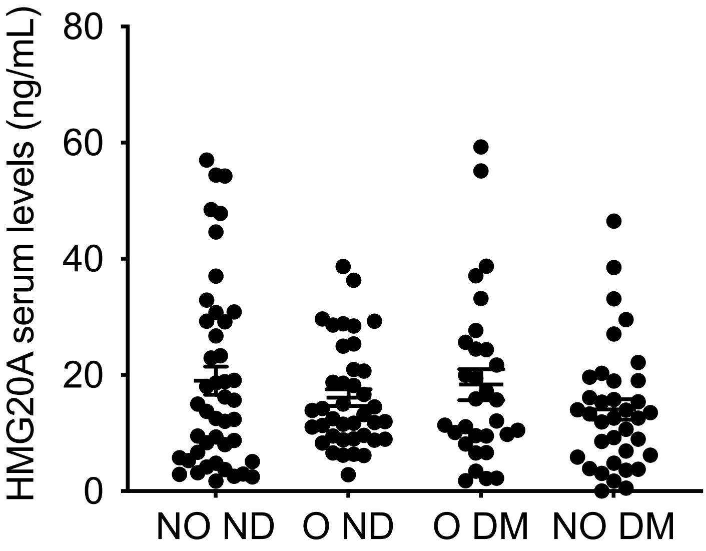

Figure S5. HMG20A serum levels. Quantification of HMG20A protein levels in serum isolated from the various groups depicted in the graph using an ELISA kit for human HMG20A. *n = 40-50* individuals per group analyzed in duplicate.

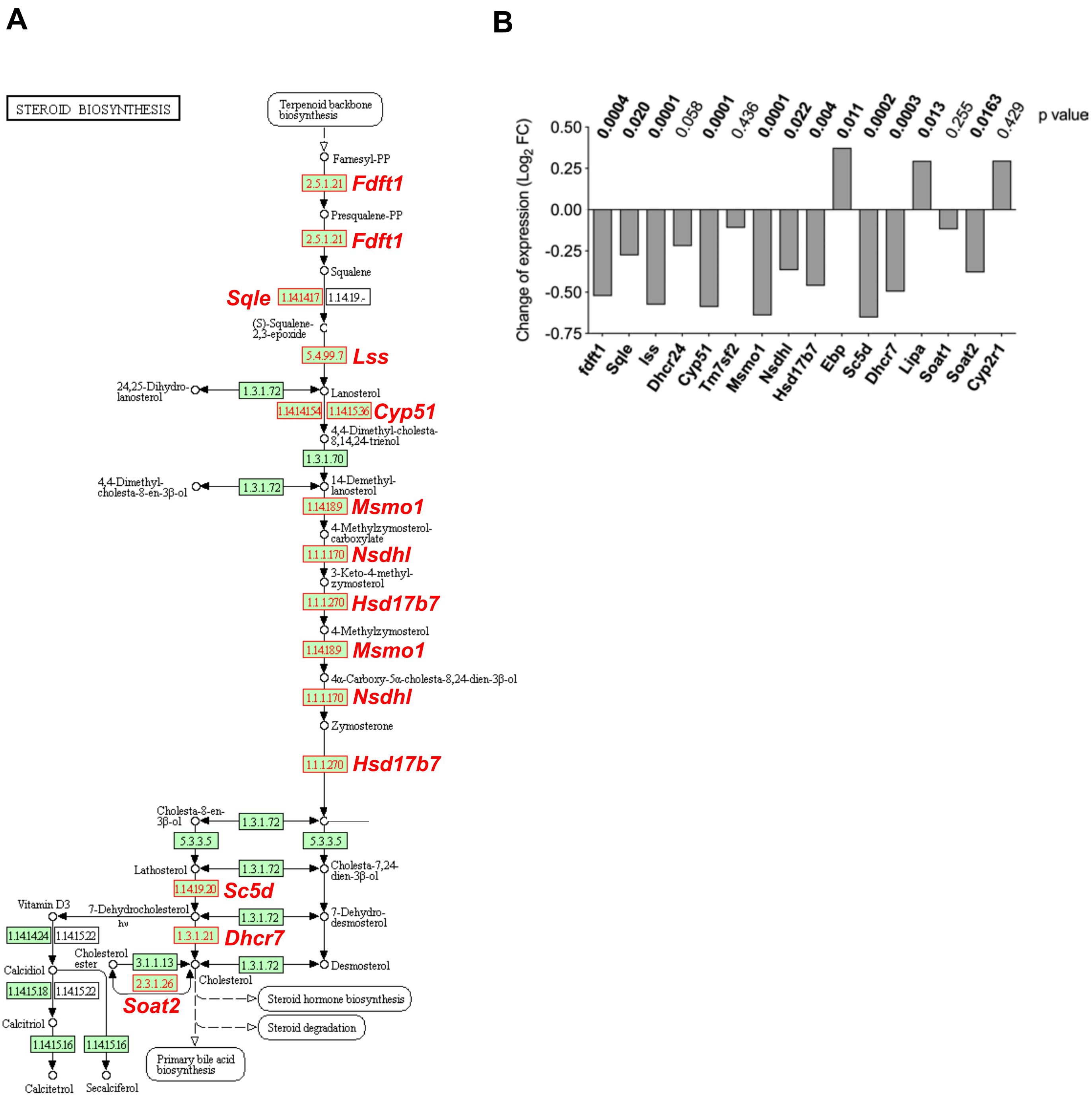

Figure S6. Regulation of steroid biosynthesis pathway after *Hmg20a* silencing in astrocytes. (**A**) **The** KEGG pathway of steroid biosynthesis showing the regulated genes after *Hmg20a* silencing. Enzymes present in mammals are highlighted in green. Enzymes whose encoding genes are downregulated after *Hmg20a* silencing are highlighted in red. *Mus musculus* gene names encoding the indicated enzyme are also included. (**B**) Bar Graph representing the relative expression levels of significantly regulated genes involved in sterol biosynthesis pathway, analyzed by LIMMA software. *p* value for each of the regulated genes is indicated above the bars. Values are referred to the expression levels detected in siCT.

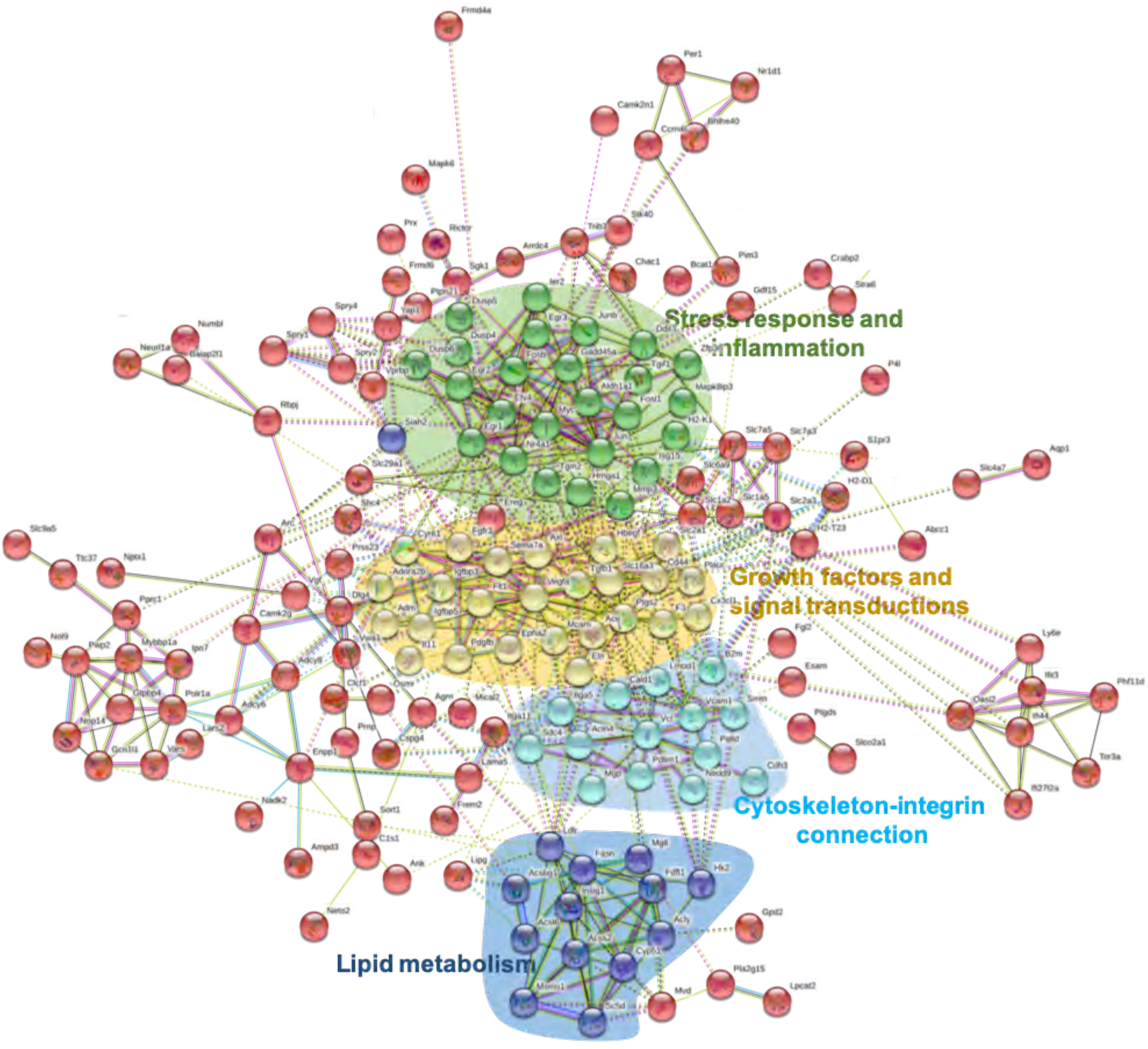

Figure S7. Main biological networks regulated by HMG20A in astrocytes. STRING cluster analysis of the 245 down-regulated genes in HMG20A repressed astrocytes. Only interacting mRNAs that were significantly repressed by siHMG20A are depicted. Criteria used were: Minimum required interaction score 0.4 and K-means clustering set to 5. The identified clusters are coloured and labelled as depicted in the figure. The solid and the dotted lines indicate connection within the same and different cluster respectively. Different color indicates different type of interactions: Cyan-from curated databases; Pink-experimentally determined; Blue-gene co-occurrence; Khaki-from text mining; Black-co-expression; Light blue-protein homology).

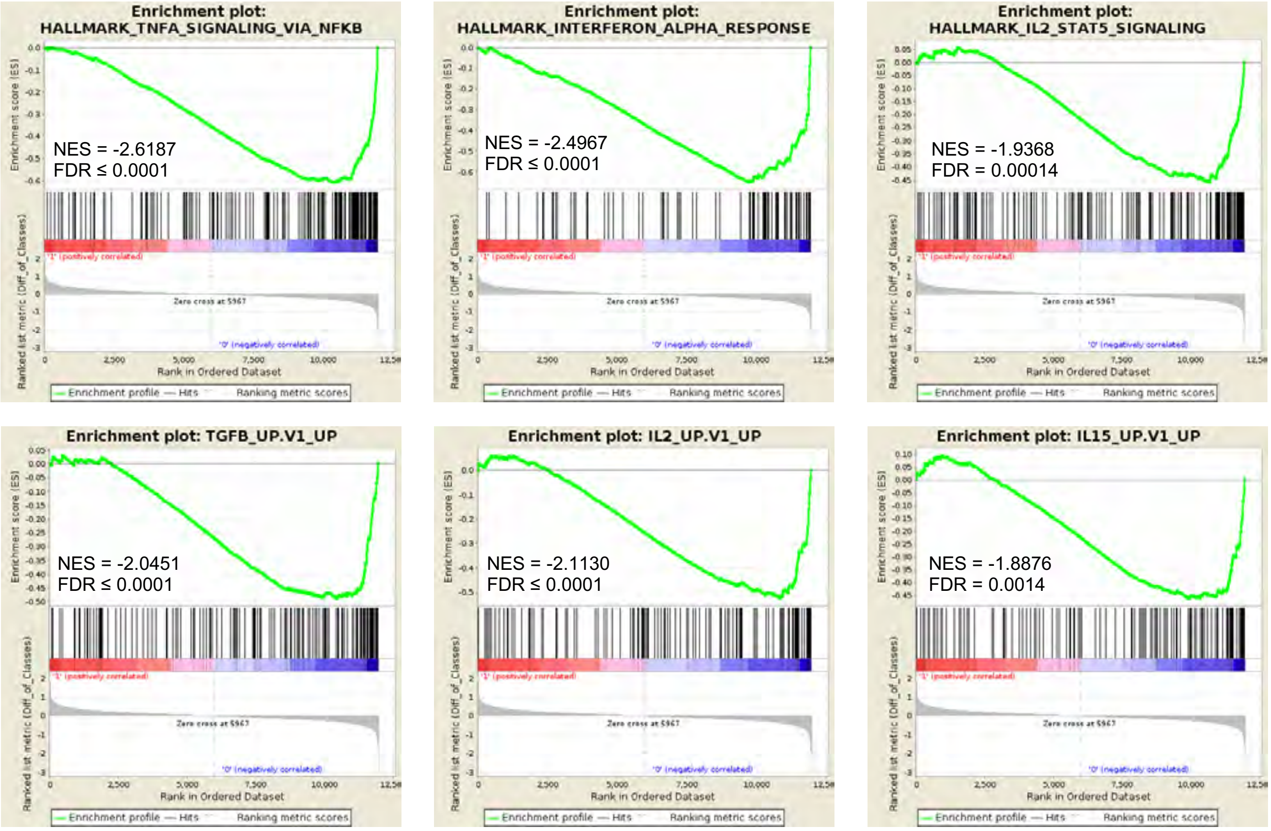

Figure S8. Pathways enriched after *Hmg20a* silencing. Enrichment plots of key inflammatory pathway enriched after *Hmg20a* silencing. The analysis was performed using the GSEA software. Normalized Enriched Score and FDR are provided within each plots.

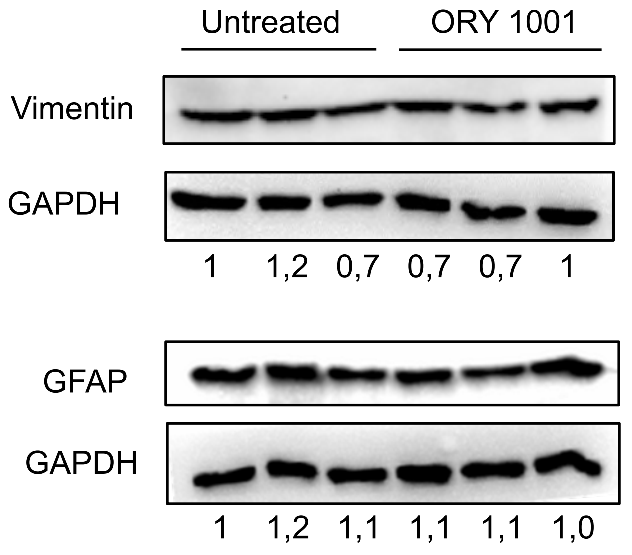

Figure S9. GFAP and VIMENTIN levels in ORY-1001-treated astrocytes. Protein levels of GFAP and VIMENTIN were assessed in whole cell extracts of astrocytes cultured or not with ORY-1001 for 72 hours. Values shown below are the relative expression levels of each protein normalized to GADPH.
